# Fertility-Preserving Treatments and Patient- and Parental Satisfaction on Fertility Counseling in a Cohort of Newly Diagnosed Boys and Girls with Childhood Hodgkin Lymphoma

**DOI:** 10.3390/cancers16112109

**Published:** 2024-05-31

**Authors:** Katja C. E. Drechsel, Irene M. IJgosse, Sofie Slaats, Lisanne Raasen, Francis S. Stoutjesdijk, Eline van Dulmen-den Broeder, W. Hamish Wallace, Auke Beishuizen, Dieter Körholz, Christine Mauz-Körholz, Michaela Cepelova, Anne Uyttebroeck, Leila Ronceray, Gertjan J. L. Kaspers, Simone L. Broer, Margreet A. Veening

**Affiliations:** 1 Emma Children’s Hospital, Amsterdam University Medical Center, Vrije Universiteit Amsterdam, Pediatric Oncology, Cancer Center Amsterdam, 1007 MB Amsterdam, The Netherlandsma.veening@prinsesmaximacentrum.nl (M.A.V.); 2 Princess Máxima Center for Pediatric Oncology, 3584 CS Utrecht, The Netherlands; 3 Cancer Center Amsterdam, Amsterdam University Medical Center, Vrije Universiteit Medical Center, 1007 MB Amsterdam, The Netherlands; 4 Department of Haematology/Oncology, Royal Hospital for Sick Children, Edinburgh EH16 4TJ, UK; 5 Department of Haematology/Oncology, Erasmus MC-Sophia Children’s Hospital, 3000 CA Rotterdam, The Netherlands; 6 Department of Pediatric Hematology and Oncology, Universitätsklinikum Giessen und Marburg GmbH, Standort Giessen—Zentrum für Kinderheilkunde und Jugendmedizin, Feulgenstr. 12, 35392 Giessen, Germanychristine.mauz-koerholz@paediat.med.uni-giessen.de (C.M.-K.); 7 Clinic for Paediatric and Adolescent Medicine, Medical Faculty of the Martin-Luther University of Halle, Ernst-Grube-Str. 40, 06120 Halle, Germany; 8 Department of Pediatric Hematology and Oncology, Faculty Hospital Motol and 2nd Medical Faculty, Charles University, V Úvalu 84, 150 06 Prague 5, Czech Republic; 9 Department of Paediatric Haematology and Oncology, KU Leuven, UZ Leuven, Herestraat 49, 3000 Leuven, Belgium; 10 Pediatric Hematology and Oncology, St. Anna Children’s Hospital, Medical University of Vienna, Kinderspitalgasse 6, A-1090 Wien, Austria; 11 Department of Reproductive Medicine & Gynecology, University Medical Center Utrecht, 3584 CX Utrecht, The Netherlands

**Keywords:** fertility counseling, fertility preservation, childhood Hodgkin lymphoma

## Abstract

**Simple Summary:**

Most children diagnosed with (classical) Hodgkin lymphoma survive, but chemotherapy and radiotherapy can harm their fertility. There are several fertility-preserving treatments available that can be used in effort to preserve reproductive ability. In this observational study, we studied how often fertility-preserving treatments were used in a cohort of children with newly diagnosed classical Hodgkin lymphoma and evaluated the patient- and treatment characteristics of those receiving such co-treatments. Furthermore, we surveyed patients and parents/guardians to gain insight into their opinion and satisfaction on the offered fertility counseling. Most patients and parents/guardians had received fertility counseling. Most participants were satisfied about the offered counseling and found it important. Concerns about (future) fertility were common. This study emphasizes the importance of fertility counseling and the consideration of fertility preservation based on the expected risk of infertility and patient characteristics. The evaluation of fertility care is important considering the impact of (in)fertility on quality of life.

**Abstract:**

Purpose: The purpose of this study is to evaluate the use of fertility-preserving (FP) treatments and fertility counseling that was offered in a cohort of newly diagnosed children with classical Hodgkin lymphoma (cHL). Methods: In this observational study, boys and girls with cHL aged ≤ 18 years with scheduled treatment according to the EuroNet-PHL-C2 protocol were recruited from 18 sites (5 countries), between January 2017 and September 2021. In 2023, a subset of Dutch participants (aged ≥ 12 years at time of diagnosis) and parents/guardians were surveyed regarding fertility counseling. Results: A total of 101 boys and 104 girls were included. Most post-pubertal boys opted for semen cryopreservation pre-treatment (85% of expected). Invasive FP treatments were occasionally chosen for patients at a relatively low risk of fertility based on scheduled alkylating agent exposure (4/5 testicular biopsy, 4/4 oocyte, and 11/11 ovarian tissue cryopreservation). A total of 17 post-menarchal girls (20%) received GnRH-analogue co-treatment. Furthermore, 33/84 parents and 26/63 patients responded to the questionnaire. Most reported receiving fertility counseling (97%/89%). Statements regarding the timing and content of counseling were generally positive. Parents and patients considered fertility counseling important (94%/87% (strongly agreed) and most expressed concerns about (their child’s) fertility (at diagnosis 69%/46%, at present: 59%/42%). Conclusion: Systematic fertility counseling is crucial for all pediatric cHL patients and their families. FP treatment should be considered depending on the anticipated risk and patient factors. We encourage the development of a decision aid for FP in pediatric oncology.

## 1. Introduction

Nowadays, classical Hodgkin lymphoma (cHL) is one of the most curable types of cancer in children, with high survival rates (>90%) attributed to advancements in therapy [1,2]. The standard treatment strategy comprises multi-agent chemotherapy, followed by radiotherapy depending on response evaluation [1]. Applied treatment modalities may impair fertility, with serious impacts on psycho-social wellbeing and quality of life in survivors [3,4,5,6]. Therefore, depending on the estimated risk of infertility after treatment, fertility preservation (FP) becomes a crucial consideration in the management of cHL in pediatric patients.

(Post-)Pubertal boys can attempt to collect semen for cryopreservation pre-treatment. For prepubertal boys, the only current option for FP is a testicular biopsy in a research setting, as it is not (yet) certain whether it will become feasible to isolate and culture spermatogonial stem cells from prepubertal testicular tissue in the near future [7,8,9]. Established FP treatments for females include the cryopreservation of oocytes or ovarian tissue (OTC), with the latter being the only available option for pre-menarchal girls. In case of planned radiotherapy to the abdominal area, ovariopexy can be applied to transpose an ovary outside of the radiation field. Furthermore, hormonal co-treatment with gonadotropin-releasing hormone analogues (GnRH-as) has been used to induce endocrine suppression and inhibit ovarian cellular turnover, which is hoped to reduce the chance of cellular destruction during treatment with gonadotoxic modalities. However, scientific evidence of the effectiveness of GnRH-as to protect the ovarian reserve is scarce and only found in breast cancer patients; therefore, guidelines do not recommend GnRH-as for clinical care [10,11,12].

The consideration of FP is typically made in accordance with established guidelines, factoring in individual risk assessments and other relevant factors such as age, severity of illness, and the timeframe available for FP procedures. According to the European PANCARE guidelines, females up to the age of 25 years with planned treatment with alkylating agent exposure at a cyclophosphamide-equivalent dose score (CED score) of ≥6000 mg/m^2^ are considered to be at high risk of infertility, while for males, this cutoff is set at ≥4000 mg/m^2^ [7,10]. Additionally, females expected to undergo pelvic radiotherapy, as well as males receiving inguinal or testicular irradiation, are also classified as being at a high risk of infertility.

Previous studies have shown that fertility counseling, by informing patients about the potential impact of treatment on reproductive ability and available FP options, leads to enhanced quality of life and less regret regarding decisions about FP [13,14,15]. Even patients with an anticipated low risk of infertility tend to harbor concerns about their future reproductive abilities, highlighting the importance of educating all patients (and parents in the setting of childhood cancer) comprehensively [14]. There is an increasing trend towards regulated, protocol-driven oncological fertility care programs [16,17]. The evaluation of the counseling that was offered and FP will be of value to further improve fertility care for future patients and survivors. We aimed to evaluate the fertility care that was provided among children with newly diagnosed cHL, by studying the overall use of FP treatments, including reasons for not offering or pursuing FP in a cohort of patients ≤ 18 years old participating in the European fertility add-on study (to the EuroNet-PHL-C2 study). Moreover, we assessed patient and parental satisfaction on fertility counseling and FP options, and potential decisional stress through a questionnaire in a subset of Dutch patients.

## 2. Methods

### 2.1. Study Design and Study Population

The present study is embedded in the fertility add-on study, an international prospective cohort study aiming to evaluate the gonadotoxic effects of the current European EuroNet-PHL-C2 treatment protocol for childhood HL, and to analyze the utilization of fertility-preserving methods within this population. The fertility add-on study has a total of 18 participating sites in 5 countries (the Netherlands, Belgium, Germany, Austria, and Czech Republic), and is an add-on study to the larger EuroNet-PHL-C2 trial (NCT02684708) [18]. Eligible participants included boys and girls with a confirmed cHL diagnosis up to the age of 18 years old, with scheduled treatment according to the C2 protocol. Patients were recruited for the fertility add-on study between January 2017 and September 2021.

To evaluate fertility counseling, Dutch participants (and their parents) who provided informed consent for follow-up research were invited in 2023 to participate in an additional survey. Patients who were deceased, had recurrence, a second malignancy, or were lost to follow-up were not invited for the survey. All patients and/or their parents/guardians were contacted via phone and in the case of verbal consent, informed consent forms as well as the questionnaire were sent to the home address of the participant. The survey consisted of two questionnaires: ‘questionnaire 1’ was designed for parents, and ‘questionnaire 2’ was designed for children aged 12 years and older at the time of cHL diagnosis. If the patient was under 12 years old at the time of cHL diagnosis, only parents/guardians were invited to participate. ‘Questionnaire 1’ could be completed by the father and/or mother or guardian of the patient; the identity of the respondent was not recorded. All returned questionnaires of patients or parents, who had a fully signed informed consent form, were included in the analysis of survey results. The fertility add-on study and additional survey (as an amendment) were approved by ethical board(s) (i.e., the international fertility add-on study was approved by ethical boards of all participating countries and the Dutch survey was approved by the ethical board of Amsterdam University Medical Center, the Netherlands). This study was conducted in accordance with good clinical practice and the Declaration of Helsinki.

### 2.2. Data Collection and Measurements

#### 2.2.1. Fertility-Preserving Treatments in the Fertility Add-On Study Cohort

Information regarding semen collection or testicular biopsy for cryopreservation in boys was obtained prior to the onset of treatment. Data on potential FP procedures in girls, including oocyte cryopreservation, OTC, and ovariopexy, as well as GnRH-a co-treatment, were collected after the completion of treatment. Reasons for not offering or abstaining from FP were also documented by the research staff on the Case Report Form (CRF) using a predefined list of response options, tailored for boys and girls (see Supplementary Table S1). It should be emphasized that the fertility add-on study was observational, and all FP treatments were offered within the framework of regular care. However, for those undergoing a testicular biopsy, FP was offered within a research setting that was independent of the fertility add-on study.

Furthermore, data on age and Tanner stage (G: genital for boys, M: mammae for girls), menarchal status and menstrual cycle (in girls), and testicular volume (in boys, measured using a Prader orchidometer) at diagnosis were extracted from medical records. Tanner stage M ≥ 2 for girls was used to define the onset of puberty, while for boys, a testicular volume ≥ 4 mL (or in case of missing data: Tanner stage G ≥ 2) was utilized. Boys at Tanner stage G 4 and/or with a testicular volume ≥ 15 mL were expected to be able to collect semen for cryopreservation. Boys who had unsuccessful semen cryopreservation due to azoospermia were still included in the semen cryopreservation FP group.

#### 2.2.2. Fertility Questionnaire

The questionnaires regarding fertility counseling and FP were designed in Dutch by the study team based on other validated (fertility)questionnaires [19,20,21,22]. Distinct versions of questionnaires were designed for parents (‘questionnaire 1’) and patients (‘questionnaire 2’, shorter version), each adapted to accommodate gender-specific considerations (i.e., to take the available FP options for either boys or girls, and potential conversation with gynecologist or urologist, into account). The questionnaires included multiple choice questions and statements about setting, content, and importance of fertility counseling, as well as potential concerns regarding (future) fertility. Parents were also asked whether FP options were discussed and/or offered, and if the decision on FP was paired with decisional stress or regret. All statements could be responded to using a scale, with answer options ranging from “completely disagree”, “disagree”, “neither disagree nor agree” (referred to as ‘neutral’ in the present paper), “agree”, to “completely agree”, or “I don’t know”. The Dutch questionnaires were approved by the ethical committee and are included in the Supplementary Materials (see Supplementary File S1).

#### 2.2.3. Other Patient Characteristics, cHL Treatment Data, and Estimated Risk of Infertility

Data regarding the cHL diagnosis, including staging and involved tumor sites, as well as treatment details and potential recurrence or second malignancies during follow-up, were extracted from the central EuroNet-PHL-C2 study-database.

Within the C2 treatment protocol, the assigned treatment and number of planned chemotherapy courses depend on the treatment level (TL), which is determined by the stage of disease and associated risk factors. TL1 denotes early-stage disease, TL2 signifies intermediate-stage disease, and TL3 indicates advanced-stage disease. All patients receive standard OEPA induction chemotherapy, containing vincristine, etoposide, prednisone, and doxorubicin. TL1 patients receive subsequent COPDAC-28 consolidation (cyclophosphamide, vincristine, prednisone, dacarbazine) or involved node radiotherapy depending on treatment response. Patients in TL2 and TL3 are randomly allocated to receive either the standard COPDAC-28 or the intensified DECOPDAC-21 consolidation course (doxorubicin, etoposide, cyclophosphamide, vincristine, prednisone, dacarbazine), with additional radiotherapy depending on treatment response. For further clarification, please refer to the C2-study protocol [18].

The estimated risk of infertility of the assigned treatment was determined by calculating the CED score of the assigned treatment and applying the previously mentioned CED score cutoff values of the PANCARE guidelines (high risk for boys ≥ 4000 mg/m^2^, girls ≥ 6000 mg/m^2^) [7,10,23]. The calculated CED score ranges between 0 and 5000 mg/m^2^ for the C2 protocol (i.e., TL1: CED score 0–1000 mg/m^2^, TL2-COPDAC-28 2000 mg/m^2^, TL2-DECOPDAC-21 2500 mg/m^2^, TL3-COPDAC-28 4000 mg/m^2^, and TL3-DECOPDAC-21 5000 mg/m^2^). Accordingly, all boys assigned to TL3 are considered at high risk of infertility, while all TL1/TL2 staged boys, as well as all girls treated for cHL according to the C2 protocol, are considered at low risk of infertility unless they receive pelvic (girls) or inguinal/testicular (boys) radiotherapy. Within this study, girls with active tumor sites in the abdominal area (i.e., mesenteric, upper/lower para-aortic, iliac, and inguinal areas) at diagnosis were considered to be at a potential risk of pelvic radiotherapy. Similarly, boys with active tumor sites in the inguinal area were considered at risk of inguinal irradiation.

### 2.3. Statistical Analysis

Baseline data were evaluated descriptively among participants of the fertility add-on study, and in subsets of Dutch patients who did and did not participate in the survey. Age, Tanner stage, testicular volume (boys), menarchal status (girls), anticipated risk of infertility based on assigned treatment, and involved tumor sites were assessed separately for patients who underwent the various available FP treatments, as well as for those who did not undergo FP. Girls who received both FP and GnRH co-treatment were included in all applicable subgroups of received FP/co-treatments. The reasons that were recorded for not undergoing semen cryopreservation (boys) or ovariopexy, OTC, or oocyte cryopreservation (girls) were descriptively examined.

Survey data of the questionnaires completed by parents and children were descriptively analyzed. The results of the statements were visually presented in figures, illustrating the distribution of answers to the statements. The time interval between the diagnosis of cHL and the completion of the survey, as well as the age at the time of the study, were calculated in years using the date of questionnaire completion (or, if unavailable, the date of signing informed consent for survey participation). If a question was left unanswered, missing data were addressed through pair-wise exclusion. Additionally, in secondary analyses, responses to questionnaires were descriptively compared based on the gender of the treated child.

All analyses were performed with IBM SPSS statistics version 28.0 (NY: IBM Corp, Released 2021). 

## 3. Results

### 3.1. Study Population

A total of 205 patients (101 boys, 104 girls) were included in the analysis on FP treatments in the fertility add-on study population. Of these, one-hundred and seven patients were treated in the Netherlands, fifty in Belgium, thirty-one in Czech Republic, thirteen in Germany, and four in Austria. The median age at diagnosis was 15.6 years (IQR 13.7;17.1) in girls and 14.8 years (IQR 11.5;16.1) in boys. Most patients were post-pubertal at the time of diagnosis (95% of girl, 72% of boys respectively), see Table 1. A total of 51 boys had advanced-stage disease; thus, these boys were assigned to TL3 and were considered to be at high risk of infertility based on their CED score, which ranged between 4000–5000 mg/m^2^. A total of twenty-eight girls (27%) had involved tumor sites in the abdominal region, of whom five girls eventually received pelvic radiotherapy (target dose 19.8 Gy). Six boys underwent radiotherapy targeted at the pelvic (iliacal) area, yet none were irradiated to the inguinal area. The expected radiotherapy dose to the testes was less than 0.5–1 Gy.

**Figure 1:**
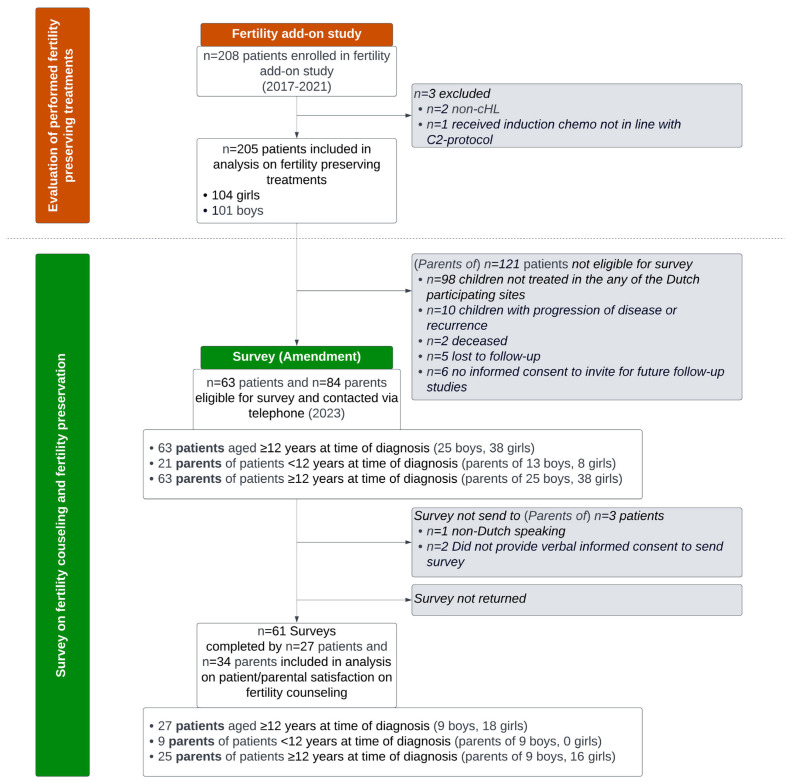
Study flowchart. cHL: classical Hodgkin lymphoma.

As depicted in the study flowchart in Figure 1, 84 parents and 63 patients (aged ≥ 12 years at time of cHL diagnosis) were considered eligible and invited for the additional Dutch fertility questionnaire. Ultimately, fifty-nine completed questionnaires, belonging to thirty-three parents (seventeen parents of boys and sixteen parents of girls) and twenty-six patients (eight boys, eighteen girls), were included in the analysis of survey results. The response rate was 39% among parents and 41% among patients. The median interval between diagnosis and the survey was 3.2–4.5 years. Information on the HL diagnosis and assigned treatment of the patients that participated in the Dutch survey is included in Table 1. There were no statistically significant differences in cHL diagnosis and treatment characteristics between the eligible Dutch patients that eventually did and did not participate in the survey.

### 3.2. Fertility Preservation in Boys

Table 2 provides a comprehensive summary of FP treatments administered in the fertility add-on study cohort. Of the one-hundred and one participating boys in the fertility add-on study, forty-eight (48%) collected semen for cryopreservation (forty-seven self-collection, one after electroejaculation under anesthesia), with a median age of 16.0 years (IQR 13.6;18.7) and median testicular volume of 20.0 mL (IQR 15.0;22.8). The youngest boy was 13.6 years old and had a testicular volume of 15.0 mL and was classified at Tanner stage G 3. Five boys (5%, three in the Netherlands, two in Belgium) underwent a testicular biopsy prior to the onset of chemotherapy; only one of them (20%) was considered to be at a high risk of infertility. The boys who underwent a testicular biopsy to preserve tissue and those who had no FP treatment were younger compared to those who collected semen, with a median age of 12.8 years (range 3.9;14.8) and 11.8 years (range 3.4;17.8), respectively.

Most of the boys in whom sperm collection was expected to be feasible based on their Tanner stage (≥4) and/or testicular volume (≥15 mL) eventually delivered semen (85%, n = 46/55). Of the remaining nine boys, one boy underwent a testicular biopsy as he was reported to physically and emotionally not be able to collect semen. Additional recorded reasons for not performing semen cryopreservation included ‘physically not able’ (n = 1), ‘emotionally not able’ (n = 1), ‘patient/parental refusal’ (n = 4), ‘not suggested by physician’ (n = 1), and ‘unknown’ (n = 1), see Supplementary Table S1.

### 3.3. Fertility Preservation in Girls

Two girls (2%) underwent ovariopexy, both of whom were assigned to TL2. Remarkably, none of these girls had a tumor site within the abdominal area and neither received radiotherapy. Four girls (4%) cryopreserved oocytes before treatment and eleven (11%) cryopreserved ovarian tissue (OTC). The median age was 16.0 years (range 15.4–17.4) among the girls who cryopreserved oocytes and 15.5 years (range 13.2–17.8) among the girls who underwent OTC. Only three (27%) patients who had an OTC were within the TL3 treatment group; the other girls had a less advanced stage of cHL and were scheduled to receive less intense chemotherapy (i.e., CED score 0–2500 mg/m^2^). GnRH-as were prescribed to 17 girls (20% of post-menarchal girls) during treatment. The recorded reasons for not performing FP often comprised ‘not suggested by physician’, ‘low-risk treatment’, and ‘patient/parental refusion’, see Supplementary Table S1. In Czech Republic, none of the patients underwent OTC or oocyte cryopreservation, as these were not considered standard procedures at the institution.

### 3.4. Fertility Counseling

Thirty-three out of the thirty-four parents who completed the fertility questionnaire were reported to have had at least one conversation about fertility. Most conversations occurred before the start of treatment; two parents reported that the first conversation about fertility was during cHL treatment. Most parents (61%, 20/33) had a conversation with their child’s treating physician, and 33% (11/33) had an additional conversation with a nurse practitioner. The parents of two patients spoke with the urologist and seven with a gynecologist. As reported by the parents of four boys and one girl, the child was not involved in the conversation about fertility because of a young age or being too sick.

Furthermore, most patients also recalled a conversation about fertility (89%, 24/27). All patients spoke with their caregiver and nine patients also specifically discussed fertility with their parents. An overview of survey data on fertility counseling is included in Table 3.

One parent and three patients reported not having had a conversation about fertility. Particularly, the parent reported that he/she would have liked to receive information about fertility and expressed concerns about their child’s fertility; the three patients reported being less concerned about their fertility (concerns at diagnosis and at present: n = 1 ‘disagree’, n = 1 ‘neutral’, n = 1 not answered), see Supplementary Table S2.

#### 3.4.1. Parental Satisfaction on Offered Counseling

**Figure 2:**
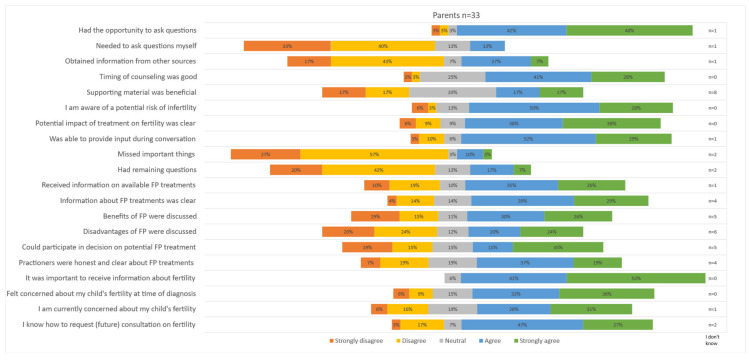
Parental satisfaction on offered fertility counseling.

Statements about fertility counseling were completed by the 32 parents (of 17 boys and 16 girls) who reported having had a conversation about fertility; the results are visually depicted in Figure 2. A comparison of the survey results between the parents of boys and parents of girls is included in Supplementary Figure S1. Most statements about the offered counseling, including the timing and content of counseling, were answered generally positively. A total of 76% of parents indicated that they had received clear information about the potential risk of infertility after their child’s treatment (38% ‘agree’, 38% ‘strongly agree’). Only 13% of parents mentioned that they had missed important things during counseling (10% ‘agree’, 3% ‘strongly agree’). Parents of girls appeared to be more likely to obtain information from other sources (54% ‘strongly agree’, versus 14% among parents of boys). Most parents reported that they were able to ask questions and provide input during the conversation (questions; 42% ‘agree’, 48% ‘strongly agree’, input: 52% ‘agree’, 29% ‘strongly agree’). A total of 61% reported that FP was discussed during the conversation. However, more than a third of parents strongly disagreed that benefits (34%) and disadvantages (44%) were discussed; particularly, the parents of boys disagreed. When compared to the parents of girls, the parents of boys seemed to feel less involved in decision making about FP when compared to the parents of girls (58% ‘strongly disagree’ among parents of boys, compared to 14% among parents of girls). Almost all parents considered information about fertility important (41% ‘agree’ and 53% ‘strongly agree’), and most were concerned about their child’s fertility (concerns at diagnosis 69% ‘strongly agree’, concerns at present: 59% ‘strongly agree’). Most parents (74%) reported to know how to request a future conversation.

#### 3.4.2. Patient Satisfaction on Offered Counseling

**Figure 3:**
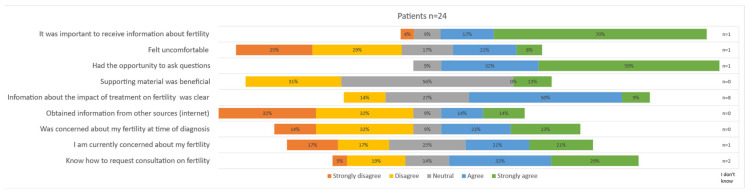
Patient satisfaction on offered fertility counseling.

All seventeen girls and seven boys that reported having had a conversation about fertility with a caregiver completed the statements about fertility counseling; the results are depicted in Figure 3. Most patients reported that the offered information about the potential impact of treatment on fertility was clear (59% ‘strongly agree’). A total of 70% ‘strongly agreed’ and 17% ‘agreed’ that it was important to receive information. Only a few patients obtained information from other sources (14% ‘agree’, 14% ‘strongly agree’). The response to the statement about whether it felt uncomfortable to talk about fertility varied widely; 25% of patients ‘strongly disagreed’, 29% ‘disagreed’, 21% ‘agreed’ and 8% ‘strongly agreed’. When comparing replies of boys and girls, it seemed that boys felt particularly uncomfortable, with 57% agreeing to feeling uncomfortable versus 18% among girls. Similarly, the rate of concerns expressed at diagnosis varied greatly among patients, with 46% responding with ‘strongly disagree’ and 46% responding with ‘strongly agree’. Boys seemed to be less concerned at diagnosis (concerns at diagnosis: 57% ‘disagree’, 15% ‘strongly disagree’), and concerns were slightly more prevalent at the time of the survey (concerns at present: 29% ‘disagree’, 14% ‘strongly disagree’). Of note, the statement about supporting material was answered with ‘neutral’ in 56% of patients. A total of 62% stated they know how to request a future conversation.

#### 3.4.3. Parental Satisfaction on Decision about Fertility Preservation

In total, seven parents of boys and eight parents of girls reported that FP was offered. As reported in Table 3, ultimately, all seven boys underwent FP; six boys delivered semen for cryopreservation, and one underwent a testicular biopsy. Among the girls, according to parental reports, ovariopexy was offered to three girls, oocyte cryopreservation to four girls, and ovarian tissue cryopreservation to five girls, all in a setting before the administration of chemotherapy. None of these girls eventually underwent FP. The reported reasons for not performing FP often included the child being too ill, high burden of FP, uncertainty about future use, and perceived low risk of infertility. Most parents reported that their child was involved in the final decision (one boy and one girl were not involved).

**Figure 4:**
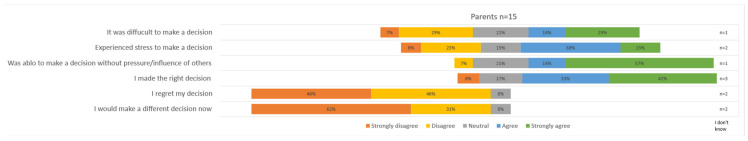
Parental satisfaction on decision regarding fertility-preserving treatment.

Particularly, the parents of girls indicated that they often found it difficult to make a decision about FP and experienced stress (difficult: 84% ‘agree’ in parents of girls vs. 17% ‘agree’ in parents of boys; decisional stress: 71% ‘agree’ in parents of girls vs. 34% ‘agree’ in parents of boys), see Figure 4. Nevertheless, the majority reported that they made the right decision (only one parent of a girl answered with ‘strongly disagree’). The statement about making the right decision was particularly positively answered by parents of boys (60% ‘strongly agree’ among parents of boys versus 29% ‘strongly agree’ among parents of girls). Nevertheless, none of the parents reported feeling regret, and nobody would have made a different decision now (of note, n = 2 answered with ‘I don’t know’).

## 4. Discussion

This study evaluated FP treatments and counseling that was offered among patients (and parents) treated for childhood cHL, who participated in a fertility study.

Based on PANCARE guidelines, semen cryopreservation should be explicitly discussed and recommended by the physician to (post-)pubertal boys, and the advice is to proceed with semen collection whenever possible, even in the case of scheduled treatment with low-dose alkylating agents [7]. Although the process of semen collection seems relatively straightforward, it should be emphasized that it can still be demanding for boys, particularly in settings where pressure is involved [24]. In our population, the vast majority of boys for whom successful semen cryopreservation was anticipated based on Tanner staging and testicular volume indeed succeeded in providing semen samples. Patient/parental refusal was scarce and abstaining from FP was rarely due to the physician not providing it.

It should be noted that cHL and its inflammatory milieu can already affect sperm quality. Based on previous studies, a low semen concentration is associated with advanced-staged disease and the presence of B symptoms [25]. It is important to inform boys in advance that their sperm quality may be impaired and attempts to freeze sperm cells could be unsuccessful in case of azoospermia. Managing disappointing outcomes can be challenging. However, stored oligozoospermic sperm samples remain viable for use in assisted reproductive technology (ART) procedures to attempt future pregnancy. Additionally, engaging in FP attempts, even if sperm preservation is not successful, may offer psychological support for acceptance in the event of male infertility.

The overall application of semen cryopreservation techniques has significantly increased over the past decades. However, the reported use rate of cryopreserved semen among oncological patients is often low (<10–15%) [26,27]. In cases with retained or recovered fertility, where males produce vital sperm cells, pregnancy may be achieved spontaneously, or fresh semen may be preferred in ART settings. Nevertheless, it is not possible to predict who will eventually become infertile due to treatment, and for males diagnosed with cancer, semen cryopreservation remains a valuable method to preserve a chance of biological offspring.

The consideration for FP becomes more challenging in boys who are physically or emotionally unable to provide semen samples, requiring a more invasive procedure for FP along with uncertainty regarding the future use of testicular tissue. If spermatogenesis has already commenced, electroejaculation can aid in obtaining ejaculate, or mature sperm cells can be extracted through testicular sperm extraction (TESE). Hopefully, ongoing research will clarify whether a testicular biopsy is deemed safe and feasible, potentially offering a solution for high-risk prepubertal cancer patients in the future.

Despite all girls being classified as low risk based on the CED score of planned treatment, and the low rate of expected FP, we still observed invasive FP methods being used in practice, even for girls receiving treatment with a CED score below 2500 mg/m^2^. Patients may be at risk of POI after treatment, yet the timing of early menopause is uncertain [28]. Nevertheless, particularly in a low-risk cohort, we can reasonably expect that the vast majority of girls will retain or resume menstruation and will not immediately experience POI after treatment (i.e., persistent acute ovarian failure). There may still be time and opportunity to freeze eggs during survivorship, before women reach the age at which their ovarian reserve is depleted. Oocyte cryopreservation is a time-consuming and invasive procedure, which is often not feasible pre-treatment in childhood cancer treatment due to a young age, severity of illness, or patients/parents’ reluctance to postpone oncological treatment. Ovarian stimulation and punction is safer and may be more bearable during survivorship. Moreover, it is unknown if and to what extend the disease itself may affect the quality of the oocytes. However, in the setting of reduced ovarian reserves following gonadotoxic treatment, the yield of oocyte vitrification may be low.

In a small subset of patients, GnRH-as were prescribed as co-treatment. This trial did not have the design to study the efficacy of GnRH-as as a gonadal protector, and additional research remains necessary. Studies on a potential difference in gonadotoxicity based on pubertal status have also not (yet) reached a consensus. Furthermore, some of the participating sites reported that specific FP treatments were not part of their standard or available procedures. It is unknown whether this was due to the (risk status of the) population or if centers may benefit from additional experience.

In cHL treatment, indication for radiotherapy is determined during treatment, depending on the response to induction chemotherapy. In the case of active tumor sites in the abdominal (girls) or inguinal (boys) area, patients may be at risk of pelvic irradiation, which can potentially be used in clinical care to further predict the ‘high-risk of infertility’ classification of patients. In the fertility add-on study cohort, two girls underwent ovariopexy, while these girls eventually did not receive radiotherapy. The ovariopexy may have been combined with another planned surgical procedure. However, it is advisable to only perform ovariopexy once the indication for radiotherapy is confirmed.

It is relevant to discuss fertility with newly diagnosed children and their families, regardless of high or low-risk classification. Both parents and children emphasized the importance of fertility counseling in the survey. Although almost everyone reported being well-informed about a potential negative impact of HL treatment on fertility, FP methods (and potential benefits and disadvantages) were not as frequently discussed, and many respondents still expressed ongoing concerns about (future) fertility. It may be beneficial to discuss fertility more than once, tailored to the phase someone is in. Fertility often tends to become an important topic during survivorship and should specifically be addressed during follow-up.

One of the statements of the survey queried the use of supportive material during counseling sessions. Parents and patients quite frequently replied to this statement with ‘don’t know’ or ‘neutral’ (50% of parents and 33% of patients, respectively). As far as we know, there is currently no standardized material available to discuss fertility in a pediatric oncology setting (in the Netherlands). Physicians often use illustrations and other visual aids at their discretion to enhance clarity during counseling sessions. Decision aids are found to be highly beneficial in providing structure to information and choices, with a significant increase in knowledge and satisfaction about decision-making [29,30,31]. We highly recommend developing a decision aid for FP specifically tailored to the childhood cancer population.

The fertility survey did not delve further into other factors that may influence the provision of fertility counseling, such as shame, inexperience, or the insecurity of the physician [32,33]. Other studies also observed that limited time, severity of illness, expected low risk of infertility, and religion can be potential barriers for not discussing fertility [31]. It is important to maintain an awareness of fertility and ensure proper training and up-to-date knowledge among all pediatric oncology health care providers. Structured fertility care, potentially through specialized research nurses, may be beneficial, as implemented in the Princess Máxima Center for Pediatric Oncology in the Netherlands [34].

### 4.1. Strengths and Limitations

This study provides an overview of the fertility care that was provided in a cohort of patients treated for cHL, also including the perspectives of patients and parents, and emphasizes the importance of fertility counseling. However, the limited power and setting of the survey within a fertility study brings certain limitations. It can reasonably be expected that all participants of a fertility study will be (more extensively) informed about fertility (although some patients and a parent still reported that they never received fertility counseling). Our participants may have had a high willingness to engage in a fertility survey, yet, they may also have stronger opinions about fertility or place greater value on counseling, when compared to other pediatric oncology populations, which could potentially have distorted the results. The counseling and FP offered were solely based on self-reporting and most patients had been diagnosed several years prior to the survey, which may introduce recall bias. Furthermore, the overall sample size and response rate was low despite personal invitation via telephone. The questionnaire was specifically tailored to Dutch cHL patients, and findings may not be applicable to counseling and expectations of parents and patients in other settings or countries. Previous studies have reported that patients and families tend to express dissatisfaction with respect to the provided information on fertility risks and options to preserve fertility [35,36]. It is relevant to conduct (repeated) larger evaluations within oncological populations to assess overall fertility care practices and patient perspectives.

This study involved a population at a relatively low risk of infertility (especially low-risk girls) in whom FP was not always offered, leading to a very limited sample size for evaluating counseling and decisions on FP. It would be particularly interesting to conduct further research within high-risk populations to further assess decisional stress and regret and potential ways to improve counseling. It is recommended to use a standardized decision regret scale to comprehensively evaluate this aspect. In addition, it would be of value to study the effectivity and contribution of FP to the likelihood of achieving parenthood after gonadotoxic treatment.

### 4.2. Summarizing Conclusions

In the fertility add-on study population, many post-pubertal boys diagnosed with cHL opted for semen cryopreservation, and invasive FP methods were occasionally chosen for patients at a relatively low risk of infertility based on the CED score of planned treatment. The studied Dutch subset of patients and their parents emphasized the importance of fertility counseling, and often expressed concerns about fertility after treatment. Overall, the participants expressed a positive attitude towards the counseling that was offered.

Fertility counseling is crucial for all patients and their families and should be systematically offered. Depending on the risk, age, and other relevant factors, it is essential to consider FP. We encourage the development of a decision aid for FP in pediatric oncology.

## Figures and Tables

**Table 1:** Baseline.

	All Patients Included in the Fertility Add-On Study		Surveys Completed by Parents (n = 34)		Surveys Completed by Patients (n = 27)	
	Girls (n = 104)	Boys (n = 101)	Parents of Girls (n = 16)	Parents of Boys (n = 18)	Girls (n = 18)	Boys (n = 9)
**HL diagnosis**						
Age at diagnosis (in years), median (IQR)	15.6 [13.7;17.1]	14.8 [11.5;16.1]	15.9 [13.7;17.1]	12.0 [9.3;17.1]	15.9 [13.8;17.3]	15.6 [13.6;17.0]
Post-pubertal at diagnosis ^a^	99 (95.2%)	73 (72.3%)	16 (100%)	9 (50.0%)	18 (100.0%)	8 (88.9%)
Assigned Treatment level						
- TL1 (early-stage disease)	18 (17.3%)	14 (13.9%)	3 (18.8%)	1 (95.6%)	3 (16.7%)	0 (0.0%)
- TL2 (intermediate-stage disease)	50 (48.1%)	36 (35.6%)	7 (43.8%)	3 (16.7%)	8 (44.4%)	1 (11.1%)
- TL3 (advanced-stage disease)	36 (34.6%)	51 (50.5%)	6 (37.5%)	14 (77.8%)	7 (38.9%)	8 (88.9%)
Anticipated high risk of infertility based on CED score of planned chemotherapy	0 (0.0%)	51 (50.5%)	0 (0.0%)	14 (77.8%)	0 (0.0%)	8 (88.9%)
Involved tumor sites in abdominal ^b^ (girls) or inguinal (boys) region	28 (26.9%)	5 (5.0%)	7 (43.8%)	2 (11.1%)	8 (44.4%)	1 (11.1%)
**Received HL treatment**						
Chemotherapy						
- 2xOEPA (*CED score 0 mg/m^2^)*	2 (1.9%)	4 (4.0%)	0 (0.0%)	0 (0.0%)	0 (0.0%)	0 (0.0%)
- 2xOEPA-1xCOPDAC-28 (*CED score 1000 mg/m^2^)*	16 (15.4%)	10 (9.9%)	3 (18.8%)	1 (5.6%)	3 (16.7%)	0 (0.0%)
- 2xOEPA-2xCOPDAC-28 (*CED score 2000 mg/m^2^)*	35 (33.7%)	20 (19.8%)	5 (31.3%0	3 (16.7%)	6 (33.3%)	1 (11.1%)
- 2xOEPA-2xDECOPDAC-21 (*CED score 2500 mg/m^2^)*	15 (14.4%)	16 (15.8%)	2 (12.5%)	0 (0.0%)	2 (11.1%)	0 (0.0%)
- 2xOEPA-4xCOPDAC-28 *(CED score 4000 mg/m^2^)*	22 (21.2%)	28 (27.7%)	3 (18.8%)	7 (38.9%)	4 (22.2%)	5 (55.6%)
- 2xOEPA-4xDECOPDAC-21 *(CED score 5000 mg/m^2^)*	14 (13.5%)	23 (22.8%)	3 (18.8%)	7 (38.9%)	3 (16.7%)	3 (33.3%)
Radiotherapy	21 (20.2%)	25 (24.8%)	5 (31.3%)	4 (22.2%)	5 (27.8%)	2 (22.2%)
Pelvic radiotherapy ^c^	5 (4.8%)	6 (5.9%)	2 (12.5%)	1 (5.6%)	2 (11.1%)	0 (0.0%)
**Questionnaire**						
Time since diagnosis (in years), median (IQR)			4.5 [3.5;5.1]	3.5 [3.0;4.2]	4.5 [3.8;5.4]	3.2 [3.0;4.2]
Age of child at time of questionnaire (in years), median (IQR)			19.8 [19.0;21.0]	16.0 [13.3;20.0]	20.0 [19.2;21.4]	20.0 [19.8;20.4]
Number of surveys completed/invited (response rate)			16/45 (36%)	18/38 (47%)	18/38 (47%)	9/25 (36%)

^a^ girls at Tanner stage Mammae ≥ 2 were considered post-pubertal. Boys were considered post-pubertal in case of testicular volume ≥ 4 mL (measured with prader orchidometer), or in case of missing data, Tanner stage Genital ≥ 2. ^b^ Abdominal region includes tumor sites in the mesenteric, upper para-aortic, lower para-aortic, iliac, and inguinal area. ^c^ none of the boys had inguinal tumor sites; expected radiotherapy dose to the testes is <0.5–1 Gy. IQR: interquartile range. TL: treatment level. OEPA: vincristine, etoposide, prednisone, and doxorubicin. COPDAC-28: cyclophosphamide, vincristine, prednisone, dacarbazine. DECOPDAC-21: doxorubicin, etoposide, cyclophosphamide, vincristine, prednisone, dacarbazine. CED score: Cyclophosphamide equivalent dose score [23].

**Table 2:** Fertility-preserving treatments.

	Males (n = 101)			Females (n = 104) ^e^				
	Semen Cryopreservation (n = 48)	Testicular Biopsy (n = 5)	No Fertility Preservation (n = 48)	Ovariopexy (n = 2)	Ovarian Tissue Cryopreservation (OTC) (n = 11)	Oocyte Cryopreservation (n = 4)	GNRH-a Co-Treatment (n = 17)	No Fertility Preservation or GNRH-a Co-Treatment (n = 76)
Age at diagnosis in years, median (range)	16.0 [13.6;18.7]	12.8 [3.9;14.8]	11.8 [3.4;17.8]	16.3 [15.4;17.2]	15.5 [13.2;17.8]	16.0 [15.4;17.4]	16.9 [13.0;18.8]	15.4 [7.2;18.0]
Tanner stage P/M, median (range)	5.0 [3.0;5.0]	2.0 [1.0;4.0]	1.0 [1.0;5.0]	3.5 [3.0;4.0]	4.0 [2.0;5.0]	5.0 [3.0;5.0]	4.0 [3.0;5.0]	4.0 [1.0;5.0]
Testicular volume (mL)	20.0 [15.0;22.8]	6.0 [5.0;12.0]	5.0 [2.0;10.0]	NA	NA	NA	NA	NA
Sperm collection expected to be feasible ^a^	46 (97.9%)	1 (20.0%)	8 (16.7%)	NA	NA	NA	NA	NA
Post-menarchal	NA	NA	NA	2 (100.0%)	9 (81.8%)	4 (100.0%)	17 (100.0%)	59 (77.6%)
Assigned Treatment level								
- TL1 (early-stage disease)	3 (6.3%)	2 (40.0%)	9 (18.8%)	0 (0.0%)	0 (0.0%)	2 (50.0%)	3 (17.6%)	13 (17.1%)
- TL2 (intermediate-stage disease)	21 (43.8%)	2 (40.0%)	13 (27.1%)	2 (100.0%)	8 (72.7%)	2 (50.0%)	11 (64.7%)	32 (42.1%)
- TL3 (advanced-stage disease)	24 (50.0%)	1 (20.0%)	26 (54.2%)	0 (0.0%)	3 (27.3%)	0 (0.0%)	3 (17.6%)	31 (40.8%)
Anticipated high risk of infertility based on assigned chemotherapy ^b^	24 (50.0%)	1 (20.0%)	26 (54.2%)	0 (0.0%)	0 (0.0%)	0 (0.0%)	0 (0.0%)	0 (0.0%)
Involved tumor sites in abdominal ^c^ (girls) or inguinal (boys) region	2 (4.2%)	0 (40.0%)	3 (6.3%)	0 (0.0%)	2 (18.2%)	0 (0.0%)	3 (17.6%)	24 (31.6%)
Pelvic radiotherapy ^d^	4 (8.3%)	0 (0.0%)	2 (4.2%)	0 (0.0%)	1 (9.1%)	0 (%)	2 (11.8%)	3 (3.9%)
Treated in country								
- The Netherlands	21	3	23	0	2	1	0	57
- Belgium	16	2	11	1	6	3	1	12
- Germany	1	0	2	1	3	0	4	6
- Czech Republic	10	0	12	0	0	0	9	0
- Austria	-	-	-	0	0	0	3	1

^a^ Sperm collection was expected in boys with Tanner stage 4/5 and/or testicular volume > 15 mL. ^b^ Cutoff to define treatment with high risk of infertility was according to current PANCARE guidelines, i.e., CED score ≥ 4000 mg/m^2^ in boys and ≥6000 mg/m^2^ in girls (with CED score = cyclophosphamide equivalent dose score) [7,10,23]. ^c^ Abdominal region includes tumor sites in the mesenteric, upper para-aortic, lower para-aortic, iliac, and inguinal area. ^d^ None of the boys had inguinal tumor sites; expected radiotherapy dose to the testes is <0.5–1 Gy. ^e^ Girls who received a FP treatment as well as hormonal co-treatment (e.g., OTC and GnRH-a) were included in all reported subgroups of received FP/co-treatments. Therefore, numbers will not add up to a total number of 104 included girls. Tanner stage P: pubis, M: mammae. TL: treatment level.

**Table 3:** Survey data on fertility counseling and fertility preservation.

	As Reported by Parents of	
	Boys (n = 18)	Girls (n = 16)
Reported fertility counseling	17 (94.4%)	16 (100.0%)
Timing counseling		
- At diagnosis	2 (11.8%)	4 (26.7%)
- After diagnosis, but before start treatment	13 (76.5%)	12 (80.0%)
- During treatment	2 (11.8%)	4 (26.7%)
- During follow-up	0 (0.0%)	2 (13.3%)
Fertility discussed by		
- Treating physician	10 (58.8%)	10 (71.4%)
- Nurse practitioner	6 (35.3%)	5 (35.7%)
- Gynecologist/urologist	2 (11.8%)	7 (50.0%)
- Missing	0	2
Child involved in conversation about fertility	13 (76.5%)	15 (93.8%)
Reasons not involved		
- Too young	3 (75.0%)	1 (100.0%)
- Too sick	0 (0.0%)	1 (100.0%)
- No interest	0 (0.0%)	0 (0.0%)
- Felt uncomfortable	0 (0.0%)	0 (0.0%)
- Missing	1	
Reported that fertility preservation was offered	7 (41.2%)	8 (50.0%)
Type of fertility preservation treatment offered/performed		
- Ovariopexy	-	3 (42.9%)/0 (0%)
- Oocyte cryopreservation	-	4 (57.1%)/0 (0%)
- Ovarian tissue cryopreservation	-	5 (71.4%)/0 (0%)
- Semen cryopreservation	6 (85.7%)/6 (100%)	-
- Testicular biopsy	1 (14.3%)/1 (100%)	-
Reported reason(s) for not performing fertility preservation		
- Too young	-	0 (0.0%)
- Too sick/high burden	-	4 (50.0%)
- Felt uncomfortable	-	0 (0.0%)
- Uncertainty about use in the future	-	2 (25.0%)
- Expected low risk	-	3 (37.5%)
Child was involved in the final decision	6 (85.7%)	7 (87.5%)
	**As reported by patients**	
	**Boys** **(n = 9)**	**Girls** **(n = 18)**
Recalls conversation about fertility	7 (77.8%)	17 (94.4%)
Fertility discussed by		
- Parents	1 (14.3%)	8 (47.1%)
- Caregiver	7 (100.0%)	17 (100.0%)

## Data Availability

Individual participant data that underlie this article cannot be shared publicly because of privacy reasons. Pseudonymized data will be shared upon reasonable request for an ethically approved study protocol, after compiling a data-sharing agreement. Data-sharing requests can be directed to ma.veening@prinsesmaximacentrum.nl.

## Supplementary Materials

The following supporting information can be downloaded at: https://www.mdpi.com/article/10.3390/cancers16112109/s1, Figure S1: Parental and patient satisfaction on fertility counseling and preservation, split for gender; Table S1: Reasons for not choosing a fertility preserving treatment as reported by research nurse on the case-report form; Table S2: Statements on fertility counseling answered by those who reported to have had no conversation about fertility; File S1: Fertility questionnaires in Dutch.
