# Supplementary material for: Fertility-Preserving Treatments and Patient- and Parental Satisfaction on Fertility Counseling in a Cohort of Newly Diagnosed Boys and Girls with Childhood Hodgkin Lymphoma
Source: Cancers (Basel). 2024 May 31;16(11):2109. doi: 10.3390/cancers16112109 (PMC11171249; doi:10.3390/cancers16112109)

Supplementary Figure S1: Parental and patient satisfaction on fertility counseling and preservation, split for gender

A. Parental satisfaction on fertility counseling

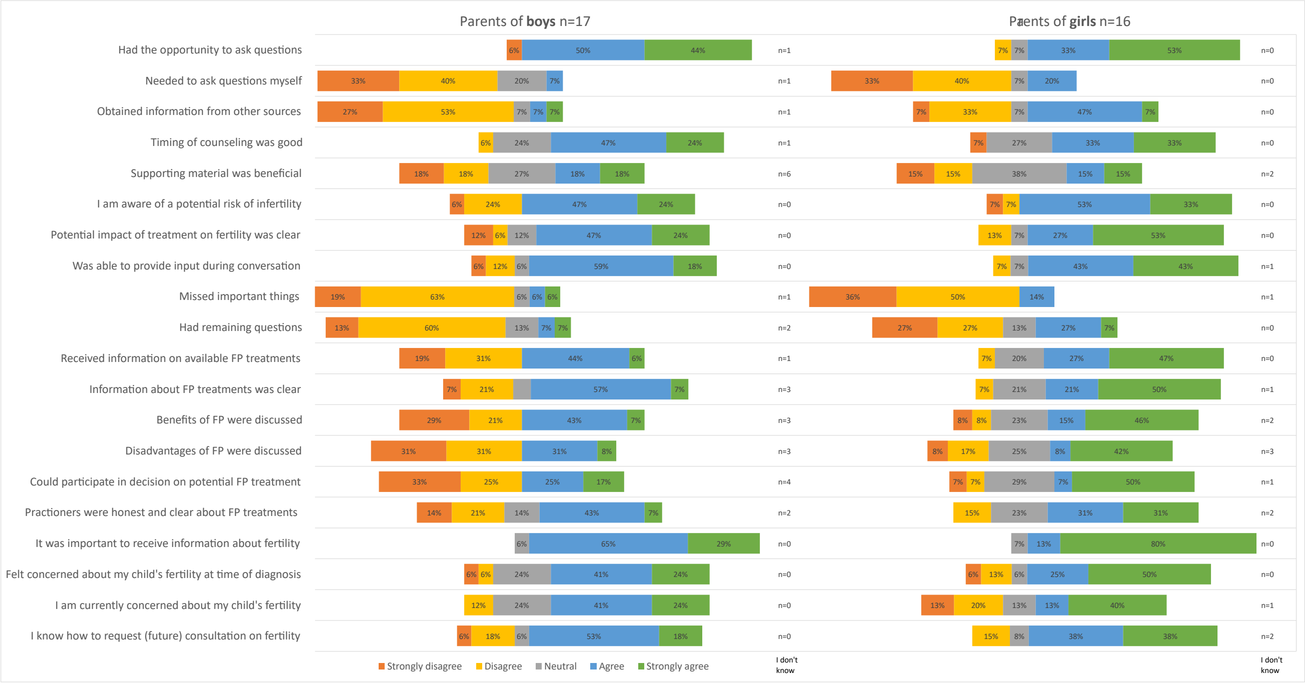

B. Patient satisfaction on fertility counseling

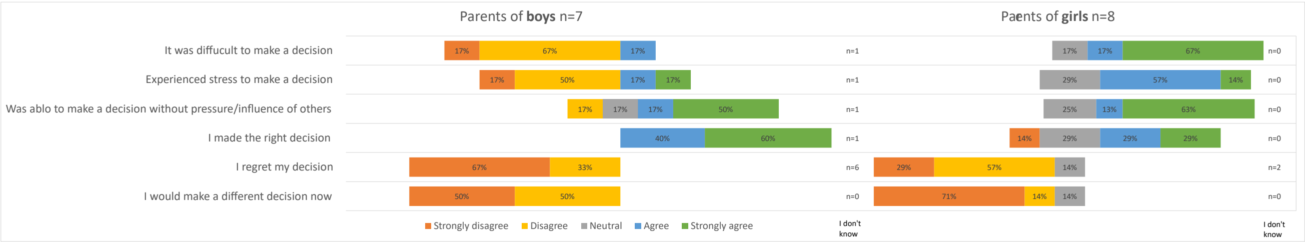

C. Parental satisfaction on decision regarding fertility preserving treatment

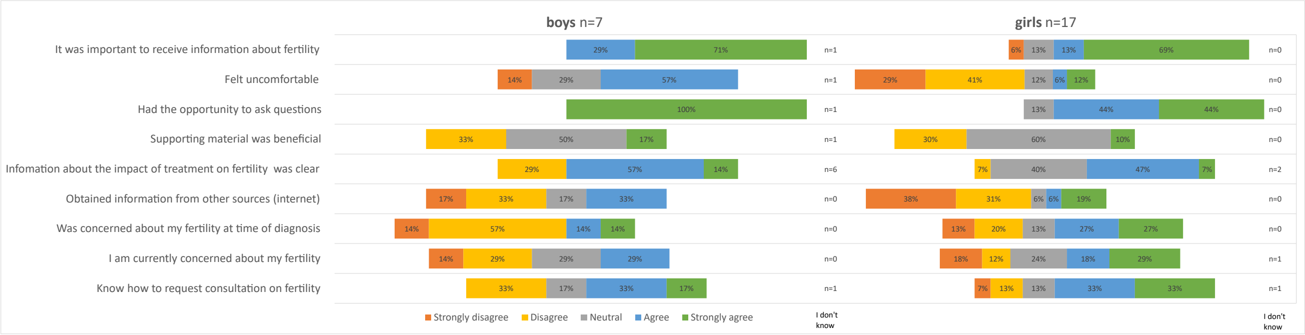

Supplement: Supplementary file 1 [file cancers-16-02109-s001.zip › Supplementary Figure S1.pdf]
