# Supplementary material for: Fertility-Preserving Treatments and Patient- and Parental Satisfaction on Fertility Counseling in a Cohort of Newly Diagnosed Boys and Girls with Childhood Hodgkin Lymphoma
Source: Cancers (Basel). 2024 May 31;16(11):2109. doi: 10.3390/cancers16112109 (PMC11171249; doi:10.3390/cancers16112109)
Supplement: Supplementary file 1 [file cancers-16-02109-s001.zip › Supplementary file S1.pdf]

Supplementary file S1: fertility questionnaires in Dutch

This supplementary file consists of the 4 different versions of the Dutch questionnaires:

1. Version female child aged ≥12 years at diagnosis: including ‘questionnaire 1’ designed for parents/guardians of girls and ‘questionnaire 2’ designed for girls
2. Version female child aged <12 years at diagnosis: including ‘questionnaire 1’ designed for parents/guardians of girls
3. Version male child aged ≥12 years at diagnosis: including ‘questionnaire 1’ designed for parents/guardians of boys and ‘questionnaire 2’ designed for boys
4. Version male child aged <12 years at diagnosis: including ‘questionnaire 1’ designed for parents/guardians of boys

EENMALIG VRAGENLIJST ONDERZOEK

Geachte ouder(s)/verzorger(s),

Uw kind kreeg de diagnose Hodgkin Lymfoom. Uw kind doet mee aan een vruchtbaarheidsstudie, de 'fertility add-on' studie die onderdeel is van de EuroNET-PHL-C2 studie. U heeft de informatie brief gelezen en aangegeven dat u mee wilt doen met een eenmalig vragenlijst onderzoek over vruchtbaarheid en de behandeling van Hodgkin.

Wij zouden ook graag een aantal vragen aan uw dochter willen stellen. Er zijn daarom 2 vragenlijsten meegestuurd. Vragenlijst 1 is voor u als ouder(s) bedoeld en vragenlijst 2 is voor uw dochter bedoeld.

Beide vragenlijsten zijn bijgevoegd bij deze brief. U kunt de vragenlijsten ingevuld retour sturen aan de onderzoekers in het Prinses Maxima Centrum via de meegestuurde antwoordenvelop.

U kunt bij vragen of opmerkingen contact opnemen met de onderzoekers via: k.c.e.drechsel-3@prinsesmaximacentrum.nl (arts-onderzoeker Katja Drechsel)

Wij willen u hartelijk danken voor uw deelname!

Dr. Margreet Veening, kinderarts-oncoloog
Drs. Katja Drechsel, arts-onderzoeker

VRAGENLIJST 1: OUDERS/VERZORGERS

Datum waarop deze vragenlijst is ingevuld: ……. - …… – 2023

VRAGENLIJST DEEL A

<u>Gesprek(ken) over vruchtbaarheid</u>

1. Is er ooit met u gesproken over de (toekomstige) vruchtbaarheid van uw kind?
   - ☐ Ja
   - ☐ Nee, ga door naar <u>deel B</u> van deze vragenlijst

2. In welke fases tijdens de behandeling is er gesproken over (toekomstige) vruchtbaarheid? (u kunt meerdere vakjes aankruisen)
   - ☐ Bij het diagnose-gesprek
   - ☐ Na het diagnose gesprek, maar voor start van de behandeling
   - ☐ Tijdens de behandeling
   - ☐ Na afronding van de behandeling

3. Met wie heeft u binnen het ziekenhuis over vruchtbaarheid gesproken? (u kunt meerdere vakjes aankruisen)
   - ☐ Behandelend arts
   - ☐ Verpleegkundig specialist
   - ☐ Gynaecoloog
   - ☐ Anders, namelijk:
     …….……
     ……….

4. Is uw dochter bij de counselingsgesprekken (die gingen over vruchtbaarheid) aanwezig geweest ?
   - ☐ Ja
   - ☐ Nee, kunt u aangeven waarom uw dochter niet betrokken is geweest bij de gesprekken die gingen over vruchtbaarheid? (u kunt meerdere vakjes aankruisen)
     - ☐ Te jong
     - ☐ Te ziek/belastend
     - ☐ Geen interesse/behoefte
     - ☐ Dit voelde ongemakkelijk
     - ☐ Anders, namelijk………………………………………………………………………………
       …….
       ………
       ………

Hieronder staan 20 uitspraken die te maken hebben met informatie en gesprekken over vruchtbaarheid. U kunt per uitspraak uit 6 opties kiezen. Geef aan hoezeer u het met deze uitspraken eens of oneens bent door het cijfer te omcirkelen bij het antwoord dat op u van toepassing is. Als u het zich niet kunt herinneren of als u geen van de antwoordopties geschikt vindt, omcirkel dan de uitspraak weet ik niet.

		Geheel mee oneens	Oneens	Niet mee oneens, niet mee eens	Eens	Geheel mee eens	Weet ik niet
1	Ik kreeg de mogelijkheid om vragen over vruchtbaarheid te stellen aan zorg-personeel (artsen, verpleegkundigen)	1	2	3	4	5	0
2	Ik heb zelf om informatie over vruchtbaarheid moeten vragen.	1	2	3	4	5	0
3	Ik heb informatie over vruchtbaarheid verkregen via andere bronnen, zoals internet, folders, andere ouders, familie, vrienden, kennissen	1	2	3	4	5	0
4	Ik vond het moment waarop gesproken werd over vruchtbaarheid een goed moment.	1	2	3	4	5	0
5	Ik vond het ondersteunende materiaal dat tijdens de uitleg gebruikt werd verduidelijkend.	1	2	3	4	5	0
6	Ik weet wat het risico voor mijn kind is op onvruchtbaarheid door de behandeling.	1	2	3	4	5	0
7	De uitleg over een mogelijk effect van de behandeling op de vruchtbaarheid was duidelijk	1	2	3	4	5	0
8	Ik heb zelf ook voldoende kunnen inbrengen tijde het gesprek/ de gesprekken over vruchtbaarheid	1	2	3	4	5	0
9	Ik heb belangrijke dingen gemist tijdens het gesprek/ de gesprekken	1	2	3	4	5	0
10	Ik had na afloop van het gesprek/de gesprekken nog steeds vragen over vruchtbaarheid.	1	2	3	4	5	0

		Geheel mee oneens	Oneens	Niet mee oneens, niet mee eens	Eens	Geheel mee eens	Weet ik niet
11	Ik heb uitleg gekregen over de mogelijkheden die er zijn om vruchtbaarheid te behouden	1	2	3	4	5	0
12	De uitleg over de behandelmogelijkheden was duidelijk	1	2	3	4	5	0
13	De voordelen van vruchtbaarheidsbehoudende behandelingen zijn besproken.	1	2	3	4	5	0
14	De nadelen van vruchtbaarheidsbehoudende behandelingen zijn besproken.	1	2	3	4	5	0
15	Er was ruimte om mee te beslissen over een eventuele behandeling voor vruchtbaarheidsbehoud.	1	2	3	4	5	0
16	Mijn behandelaars waren eerlijk en duidelijk over wat ik kon verwachten van een vruchtbaarheidsbehandeling.	1	2	3	4	5	0
17	Ik vond het belangrijk om informatie over vruchtbaarheid te krijgen	1	2	3	4	5	0
18	Toen ik de diagnose en behandeling hoorde, maakte ik mij zorgen om de vruchtbaarheid van mijn kind	1	2	3	4	5	0
19	Ik maak me op dit moment zorgen om de vruchtbaarheid van mijn kind	1	2	3	4	5	0
20	Als ik in de toekomst vragen heb over de vruchtbaarheid van mijn kind, weet ik hoe ik opnieuw een gesprek kan aanvragen.	1	2	3	4	5	0

De vragenlijst gaat door op de volgende bladzijde

Vruchtbaarheidsbehoud

5. Is er een mogelijkheid aangeboden voor een behandeling voor behoud van vruchtbaarheid?
   - ☐ Ja
   - ☐ Nee, einde van de vragenlijst

6. Wanneer werd deze vruchtbaarheidsbehoudende behandeling aangeboden?
   - ☐ Voor start van de chemotherapie
   - ☐ Tijdens de chemotherapie
   - ☐ Voor start van de bestraling
   - ☐ Na afronding van de behandeling voor Hodgkin (tijdens de follow-up)

7. Welke vruchtbaarheidsbehoudende behandeling werd er aangeboden?
   - ☐ Operatief verplaatsen van de eierstok/eierstokken binnen de buikholte, zodat de eierstokken buiten het bestralingsgebied vallen (Ovariopexie, ovariele transpositie))
   - ☐ Eicellen invriezen (oocyten cryopreservatie)
   - ☐ Eierstok weefsel invriezen (ovarian tissue cryopreservation)

8. Heeft u er uiteindelijk voor gekozen om deze vruchtbaarheidsbehoudende behandeling(en) te laten plaatsvinden?
   - ☐ Ja
   - ☐ Nee, wilt u aangeven waarom u ervoor koos om geen behandeling te laten plaatsvinden? (u kunt meerdere vakjes aankruisen)
     - ☐ Te ziek/belastend
     - ☐ Dit voelde ongemakkelijk
     - ☐ Onzekerheden over het gebruik in de toekomst
     - ☐ Anders, namelijk……………………………………………………………………………
       ……………………………………………………………………………………………………
       ……………………………………………………………………………………………………
       ……………………………………………………………………………………………………

9. Heeft uw kind mee besloten of er een vruchtbaarheidsbehoudende behandeling zou plaatsvinden?
   - ☐ Ja
   - ☐ Nee

De vragenlijst gaat door op de volgende bladzijde

Kunt u aangeven in hoeverre u het eens bent met onderstaande stellingen? U kunt per uitspraak weer uit 6 opties kiezen. Geef aan hoezeer u het met deze uitspraken eens of oneens bent door het cijfer te omcirkelen bij het antwoord dat op u van toepassing is. Op deze manier geeft u aan wat u vindt van de beslissing die u genomen heeft. Als u het zich niet kunt herinneren of als u geen van de antwoordopties geschikt vindt, omcirkel dan de uitspraak weet ik niet.

		Geheel mee oneens	Oneens	Niet mee oneens, niet mee eens	Eens	Geheel mee eens	Weet ik niet
1	Ik vond het moeilijk om een keuze te moeten maken	1	2	3	4	5	0
2	Ik ervaarde stress voor het maken van een keuze	1	2	3	4	5	0
3	Ik heb kunnen kiezen zonder druk of beïnvloeding van anderen.	1	2	3	4	5	0
4	Ik heb de juiste beslissing gemaakt over vruchtbaarheidsbehoud.	1	2	3	4	5	0
5	Ik heb spijt van de beslissing die ik heb gemaakt	1	2	3	4	5	0
6	Ik zou nu een andere beslissing maken.	1	2	3	4	5	0

Dit is het einde van vragenlijst deel A. Deel B van de vragenlijst hoeft alleen ingevuld te worden als er geen gesprek over vruchtbaarheid is geweest

VRAGENLIJST DEEL B

Dit deel van de vragenlijst hoeft u alleen in te vullen als er geen gesprek over vruchtbaarheid is geweest. Als er wel een gesprek over vruchtbaarheid is geweest en u vragenlijst deel A heeft ingevuld, bent u klaar met deze vragenlijst.

Als er geen gesprek over vruchtbaarheid is geweest

Hieronder staan 5 uitspraken die te maken hebben met vruchtbaarheid. U kunt per uitspraak uit 6 opties kiezen. Geef aan hoezeer u het met deze uitspraken eens of oneens bent door het cijfer te omcirkelen bij het antwoord dat op u van toepassing is. Als u het zich niet kunt herinneren of als u geen van de antwoordopties geschikt vindt, omcirkel dan de uitspraak weet ik niet.

		Geheel mee oneens	Oneens	Niet mee oneens, niet mee eens	Eens	Geheel mee eens	Weet ik niet
1	Ik had graag informatie over vruchtbaarheid willen krijgen van betrokken zorg-personeel (artsen, verpleegkundigen)	1	2	3	4	5	0
2	Ik heb informatie over vruchtbaarheid verkregen via andere bronnen, zoals internet, andere ouders, familie, vrienden, kennissen	1	2	3	4	5	0
3	Toen ik de diagnose en behandeling hoorde, maakte ik mij zorgen om de vruchtbaarheid van mijn kind	1	2	3	4	5	0
4	Ik maak me op dit moment zorgen om de vruchtbaarheid van mijn kind	1	2	3	4	5	0
5	Ik weet hoe ik een gesprek kan aanvragen als ik (in de toekomst) vragen heb over de vruchtbaarheid van mijn kind	1	2	3	4	5	0

Dit is het einde van de vragenlijst. Hartelijk dank voor het invullen!

VRAGENLIJST 2:

Beste ……………………………….,

Je bent behandeld voor een Hodgkin Lymfoom en je doet mee aan een studie waarbij onderzocht wordt wat de gevolgen zijn van de behandeling van Hodgkin voor de vruchtbaarheid (het krijgen van kinderen).

Wij willen met deze vragenlijst onderzoeken hoe je de gesprekken die gingen over vruchtbaarheid vond. We hebben je ouder(s) ook vragen gesteld over deze gesprekken.

De vragenlijst mag samen met de vragenlijst die ingevuld is door je ouder(s) naar ons toe gestuurd worden via de post.

Wij willen je heel erg bedanken voor het invullen van deze vragenlijst!

Datum waarop deze vragenlijst is ingevuld: ……. - …… – 2023

VRAGENLIJST DEEL A

Gesprek(ken) over vruchtbaarheid

1. Is er voor zover je je kunt herinneren ooit met jou gesproken over vruchtbaarheid?
   - ☐ Ja
   - ☐ Nee, ga door naar deel B van deze vragenlijst

2. Wie heeft er met jougesproken over vruchtbaarheid (je mag meerdere opties aanvinken)?
   - ☐ Ouders
   - ☐ Dokter of verpleegkundige (of een andere zorgverlener)

De vragenlijst gaat door op de volgende bladzijde

Hieronder staan 9 uitspraken die gaan over vruchtbaarheid. Je kan per uitspraak kiezen uit 6 antwoord-opties. Omcirkel het antwoord dat voor jou het beste bij de uitspraak past.

Als je je niet kunt herinneren hoe dit was of als je geen van de antwoordopties geschikt vindt, omcirkel dan de uitspraak weet ik niet

		Geheel mee oneens	Oneens	Niet mee oneens, niet mee eens	Eens	Geheel mee eens	Weet ik niet
1	Ik vond het belangrijk om informatie over vruchtbaarheid te krijgen	1	2	3	4	5	0
2	Ik vond het ongemakkelijk om te praten over mijn vruchtbaarheid	1	2	3	4	5	0
3	Ik kon vragen stellen aan artsen en/of verpleegkundigen	1	2	3	4	5	0
4	Ik vond het ondersteunende materiaal (zoals foto's of video's) dat tijdens de uitleg gebruikt werd verduidelijkend.	1	2	3	4	5	0
5	Ik heb duidelijke uitleg gekregen over mijn vruchtbaarheid	1	2	3	4	5	0
6	Ik heb informatie over vruchtbaarheid opgezocht op internet	1	2	3	4	5	0
7	Toen ik de diagnose en behandeling hoorde, maakte ik mij zorgen om mijn vruchtbaarheid	1	2	3	4	5	0
8	Ik maak me op dit moment zorgen om mijn vruchtbaarheid	1	2	3	4	5	0
9	Als ik in de toekomst vragen heb over mijn vruchtbaarheid, weet ik hoe ik een gesprek kan aanvragen.	1	2	3	4	5	0

Dit is het einde van vragenlijst deel A. Deel B van de vragenlijst hoeft alleen ingevuld te worden als er niet met jou gesproken is over vruchtbaarheid

VRAGENLIJST DEEL B

Dit deel van de vragenlijst hoeft je alleen in te vullen als er niet met jou gesproken is over vruchtbaarheid. Als er wel een gesprek over vruchtbaarheid is geweest, en je hebt vragenlijst deel A ingevuld, dan ben je klaar met deze vragenlijst.

Als er geen gesprek over vruchtbaarheid is geweest

Hieronder staan 5 uitspraken die te maken hebben met vruchtbaarheid. Je kan per uitspraak kiezen uit 6 antwoord-opties. Omcirkel het antwoord dat voor jou het beste bij de uitspraak past.

Als je je niet kunt herinneren hoe dit was of als je geen van de antwoordopties geschikt vindt, omcirkel dan de uitspraak weet ik niet

		Geheel mee oneens	Oneens	Niet mee oneens, niet mee eens	Eens	Geheel mee eens	Weet ik niet
1	Ik had het graag informatie willen krijgen over vruchtbaarheid	1	2	3	4	5	0
2	Ik heb informatie over vruchtbaarheid opgezocht op internet	1	2	3	4	5	0
3	Toen ik de diagnose en behandeling hoorde, maakte ik mij zorgen om mijn vruchtbaarheid	1	2	3	4	5	0
4	Ik maak me op dit moment zorgen om mijn vruchtbaarheid	1	2	3	4	5	0
5	Als ik in de toekomst vragen heb over mijn vruchtbaarheid, weet ik hoe ik een gesprek kan aanvragen.	1	2	3	4	5	0

Dit is het einde van de vragenlijst. Bedankt voor het invullen!

EENMALIG VRAGENLIJST ONDERZOEK

Geachte ouder(s),

Uw kind kreeg de diagnose Hodgkin Lymfoom. Uw kind doet mee aan een vruchtbaarheidsstudie, de ‘fertility add-on’ studie die onderdeel is van de EuroNET-PHL-C2 studie. U heeft de informatie brief gelezen en aangegeven dat u mee wilt doen met een eenmalig vragenlijst onderzoek over vruchtbaarheid en de behandeling van Hodgkin.

De vragenlijst is bijgevoegd bij deze brief. U kunt deze ingevuld retour sturen aan de onderzoekers in het Prinses Maxima Centrum via de meegestuurde antwoordenvelop.

U kunt bij vragen of opmerkingen contact opnemen met de onderzoekers via:
k.c.e.drechsel-3@prinsesmaximacentrum.nl (arts-onderzoeker Katja Drechsel)

Wij willen u hartelijk danken voor uw deelname!

Dr. Margreet Veening, kinderarts-oncoloog
Drs. Katja Drechsel, arts-onderzoeker

VRAGENLIJST: OUDERS/VERZORGERS

Datum waarop deze vragenlijst is ingevuld: ……. - …… – 2023

VRAGENLIJST DEEL A

Gesprek(ken) over vruchtbaarheid

1. Is er ooit met u gesproken over de (toekomstige) vruchtbaarheid van uw kind?
   - ☐ Ja
   - ☐ Nee, ga door naar deel B van deze vragenlijst

2. In welke fases tijdens de behandeling is er gesproken over (toekomstige) vruchtbaarheid? (u kunt meerdere vakjes aankruisen)
   - ☐ Bij het diagnose-gesprek
   - ☐ Na het diagnose gesprek, maar voor start van de behandeling
   - ☐ Tijdens de behandeling
   - ☐ Na afronding van de behandeling

3. Met wie heeft u binnen het ziekenhuis over vruchtbaarheid gesproken? (u kunt meerdere vakjes aankruisen)
   - ☐ Behandelend arts
   - ☐ Verpleegkundig specialist
   - ☐ Gynaecoloog
   - ☐ Anders, namelijk:
     ………………………………………………………………………………………………………
     ……………………………………………………………………………………………………….

4. Is uw dochter bij de counselingsgesprekken (die gingen over vruchtbaarheid) aanwezig geweest ?
   - ☐ Ja
   - ☐ Nee, kunt u aangeven waarom uw dochter niet betrokken is geweest bij de gesprekken die gingen over vruchtbaarheid? (u kunt meerdere vakjes aankruisen)
     - ☐ Te jong
     - ☐ Te ziek/belastend
     - ☐ Geen interesse/behoefte
     - ☐ Dit voelde ongemakkelijk
     - ☐ Anders, namelijk………………………………………………………………………
       ……………………………………………………………………………………………….
       ………………………………………………………………………………………………
       ………………………………………………………………………………………………

Hieronder staan 20 uitspraken die te maken hebben met informatie en gesprekken over vruchtbaarheid. U kunt per uitspraak uit 6 opties kiezen. Geef aan hoezeer u het met deze uitspraken eens of oneens bent door het cijfer te omcirkelen bij het antwoord dat op u van toepassing is. Als u het zich niet kunt herinneren of als u geen van de antwoordopties geschikt vindt, omcirkel dan de uitspraak weet ik niet.

		Geheel mee oneens	Oneens	Niet mee oneens, niet mee eens	Eens	Geheel mee eens	Weet ik niet
1	Ik kreeg de mogelijkheid om vragen over vruchtbaarheid te stellen aan zorg-personeel (artsen, verpleegkundigen)	1	2	3	4	5	0
2	Ik heb zelf om informatie over vruchtbaarheid moeten vragen.	1	2	3	4	5	0
3	Ik heb informatie over vruchtbaarheid verkregen via andere bronnen, zoals internet, folders, andere ouders, familie, vrienden, kennissen	1	2	3	4	5	0
4	Ik vond het moment waarop gesproken werd over vruchtbaarheid een goed moment.	1	2	3	4	5	0
5	Ik vond het ondersteunende materiaal dat tijdens de uitleg gebruikt werd verduidelijkend.	1	2	3	4	5	0
6	Ik weet wat het risico voor mijn kind is op onvruchtbaarheid door de behandeling.	1	2	3	4	5	0
7	De uitleg over een mogelijk effect van de behandeling op de vruchtbaarheid was duidelijk	1	2	3	4	5	0
8	Ik heb zelf ook voldoende kunnen inbrengen tijdens het gesprek/ de gesprekken over vruchtbaarheid	1	2	3	4	5	0
9	Ik heb belangrijke dingen gemist tijdens het gesprek/ de gesprekken	1	2	3	4	5	0
10	Ik had na afloop van het gesprek/de gesprekken nog steeds vragen over vruchtbaarheid.	1	2	3	4	5	0

		Geheel mee oneens	Oneens	Niet mee oneens, niet mee eens	Eens	Geheel mee eens	Weet ik niet
11	Ik heb uitleg gekregen over de mogelijkheden die er zijn om vruchtbaarheid te behouden	1	2	3	4	5	0
12	De uitleg over de behandelmogelijkheden was duidelijk	1	2	3	4	5	0
13	De voordelen van vruchtbaarheidsbehoudende behandelingen zijn besproken.	1	2	3	4	5	0
14	De nadelen van vruchtbaarheidsbehoudende behandelingen zijn besproken.	1	2	3	4	5	0
15	Er was ruimte om mee te beslissen over een eventuele behandeling voor vruchtbaarheidsbehoud.	1	2	3	4	5	0
16	Mijn behandelaars waren eerlijk en duidelijk over wat ik kon verwachten van een vruchtbaarheidsbehandeling.	1	2	3	4	5	0
17	Ik vond het belangrijk om informatie over vruchtbaarheid te krijgen	1	2	3	4	5	0
18	Toen ik de diagnose en behandeling hoorde, maakte ik mij zorgen om de vruchtbaarheid van mijn kind	1	2	3	4	5	0
19	Ik maak me op dit moment zorgen om de vruchtbaarheid van mijn kind	1	2	3	4	5	0
20	Als ik in de toekomst vragen heb over de vruchtbaarheid van mijn kind, weet ik hoe ik opnieuw een gesprek kan aanvragen.	1	2	3	4	5	0

De vragenlijst gaat door op de volgende bladzijde

Vruchtbaarheidsbehoud

5. Is er een mogelijkheid aangeboden voor een behandeling voor behoud van vruchtbaarheid?
    - ☐ Ja
    - ☐ Nee, einde van de vragenlijst

6. Wanneer werd deze vruchtbaarheidsbehoudende behandeling aangeboden?
    - ☐ Voor start van de chemotherapie
    - ☐ Tijdens de chemotherapie
    - ☐ Voor start van de bestraling
    - ☐ Na afronding van de behandeling voor Hodgkin (tijdens de follow-up)

7. Welke vruchtbaarheidsbehoudende behandeling werd er aangeboden? (u kunt meerdere vakjes aankruisen)
    - ☐ Operatief verplaatsen van de eierstok/eierstokken binnen de buikholte, zodat de eierstokken buiten het bestralingsgebied vallen (Ovariopexie, ovariele transpositie))
    - ☐ Eicellen invriezen (oocyten cryopreservatie)
    - ☐ Eierstok weefsel invriezen (ovarian tissue cryopreservation)

8. Heeft u er uiteindelijk voor gekozen om deze vruchtbaarheidsbehoudende behandeling(en) te laten plaatsvinden?
    - ☐ Ja
    - ☐ Nee, wilt u aangeven waarom u ervoor koos om geen behandeling te laten plaatsvinden? (u kunt meerdere vakjes aankruisen)
        - ☐ Te ziek/belastend
        - ☐ Dit voelde ongemakkelijk
        - ☐ Onzekerheden over het gebruik in de toekomst
        - ☐ Anders, namelijk………………………………………………………………………………
          ……
          ……
          ……

9. Heeft uw kind mee besloten of er een vruchtbaarheidsbehoudende behandeling zou plaatsvinden?
    - ☐ Ja
    - ☐ Nee

De vragenlijst gaat door op de volgende bladzijde

Kunt u aangeven in hoeverre u het eens bent met onderstaande stellingen? U kunt per uitspraak weer uit 6 opties kiezen. Geef aan hoezeer u het met deze uitspraken eens of oneens bent door het cijfer te omcirkelen bij het antwoord dat op u van toepassing is. Op deze manier geeft u aan wat u vindt van de beslissing die u genomen heeft. Als u het zich niet kunt herinneren of als u geen van de antwoordopties geschikt vindt, omcirkel dan de uitspraak weet ik niet.

		Geheel mee oneens	Oneens	Niet mee oneens, niet mee eens	Eens	Geheel mee eens	Weet ik niet
1	Ik vond het moeilijk om een keuze te moeten maken	1	2	3	4	5	0
2	Ik ervaarde stress voor het maken van een keuze	1	2	3	4	5	0
3	Ik heb kunnen kiezen zonder druk of beïnvloeding van anderen.	1	2	3	4	5	0
4	Ik heb de juiste beslissing gemaakt over vruchtbaarheidsbehoud.	1	2	3	4	5	0
5	Ik heb spijt van de beslissing die ik heb gemaakt	1	2	3	4	5	0
6	Ik zou nu een andere beslissing maken.	1	2	3	4	5	0

Dit is het einde van vragenlijst deel A. Deel B van de vragenlijst hoeft alleen ingevuld te worden als er <u>geen</u> gesprek over vruchtbaarheid is geweest

VRAGENLIJST DEEL B

Dit deel van de vragenlijst hoeft u alleen in te vullen als er geen gesprek over vruchtbaarheid is geweest. Als er wel een gesprek over vruchtbaarheid is geweest en u vragenlijst deel A heeft ingevuld, bent u klaar met deze vragenlijst.

Als er geen gesprek over vruchtbaarheid is geweest

Hieronder staan 5 uitspraken die te maken hebben met vruchtbaarheid. U kunt per uitspraak uit 6 opties kiezen. Geef aan hoezeer u het met deze uitspraken eens of oneens bent door het cijfer te omcirkelen bij het antwoord dat op u van toepassing is. Als u het zich niet kunt herinneren of als u geen van de antwoordopties geschikt vindt, omcirkel dan de uitspraak weet ik niet.

		Geheel mee oneens	Oneens	Niet mee oneens, niet mee eens	Eens	Geheel mee eens	Weet ik niet
1	Ik had graag informatie over vruchtbaarheid willen krijgen van betrokken zorg-personeel (artsen, verpleegkundigen)	1	2	3	4	5	0
2	Ik heb informatie over vruchtbaarheid verkregen via andere bronnen, zoals internet, andere ouders, familie, vrienden, kennissen	1	2	3	4	5	0
3	Toen ik de diagnose en behandeling hoorde, maakte ik mij zorgen om de vruchtbaarheid van mijn kind	1	2	3	4	5	0
4	Ik maak me op dit moment zorgen om de vruchtbaarheid van mijn kind	1	2	3	4	5	0
5	Ik weet hoe ik een gesprek kan aanvragen als ik (in de toekomst) vragen heb over de vruchtbaarheid van mijn kind	1	2	3	4	5	0

Dit is het einde van de vragenlijst. Hartelijk dank voor het invullen!

EENMALIG VRAGENLIJST ONDERZOEK

Geachte ouder(s)/verzorger(s),

Uw kind kreeg de diagnose Hodgkin Lymfoom. Uw kind doet mee aan een vruchtbaarheidsstudie, de 'fertility add-on' studie die onderdeel is van de EuroNET-PHL-C2 studie. U heeft de informatie brief gelezen en aangegeven dat u mee wilt doen met een eenmalig vragenlijst onderzoek over vruchtbaarheid en de behandeling van Hodgkin.

Wij zouden ook graag een aantal vragen aan uw zoon willen stellen. Er zijn daarom 2 vragenlijsten meegestuurd. Vragenlijst 1 is voor u als ouder(s) bedoeld en vragenlijst 2 is voor uw zoon bedoeld.

Beide vragenlijsten zijn bijgevoegd bij deze brief. U kunt de vragenlijsten ingevuld retour sturen aan de onderzoekers in het Prinses Maxima Centrum via de meegestuurde antwoordenvelop.

U kunt bij vragen of opmerkingen contact opnemen met de onderzoekers via:
k.c.e.drechsel-3@prinsesmaximacentrum.nl (arts-onderzoeker Katja Drechsel)

Wij willen u hartelijk danken voor uw deelname!

Dr. Margreet Veening, kinderarts-oncoloog
Drs. Katja Drechsel, arts-onderzoeker

VRAGENLIJST 1: OUDERS/VERZORGERS

Datum waarop deze vragenlijst is ingevuld: ……. - …... – 2023

VRAGENLIJST DEEL A

Gesprek(ken) over vruchtbaarheid

1. Is er ooit met u gesproken over de (toekomstige) vruchtbaarheid van uw kind?
   - ☐ Ja
   - ☐ Nee, ga door naar deel B van deze vragenlijst

2. In welke fases tijdens de behandeling is er gesproken over (toekomstige) vruchtbaarheid? (u kunt meerdere vakjes aankruisen)
   - ☐ Bij het diagnose-gesprek
   - ☐ Na het diagnose gesprek, maar voor start van de behandeling
   - ☐ Tijdens de behandeling
   - ☐ Na afronding van de behandeling

3. Met wie heeft u binnen het ziekenhuis over vruchtbaarheid gesproken? (u kunt meerdere vakjes aankruisen)
   - ☐ Behandelend arts
   - ☐ Verpleegkundig specialist
   - ☐ Uroloog
   - ☐ Anders, namelijk:
     ……
     ……

4. Is uw zoon bij de counselingsgesprekken (die gingen over vruchtbaarheid) aanwezig geweest ?
   - ☐ Ja
   - ☐ Nee, kunt u aangeven waarom uw zoon niet betrokken is geweest bij de gesprekken die gingen over vruchtbaarheid? (u kunt meerdere vakjes aankruisen)
     - ☐ Te jong
     - ☐ Te ziek/belastend
     - ☐ Geen interesse/behoefte
     - ☐ Dit voelde ongemakkelijk
     - ☐ Anders, namelijk………………………………………………………………………
       ………………………………………………………………………………………………
       ………………………………………………………………………………………………
       ………………………………………………………………………………………………

Hieronder staan 20 uitspraken die te maken hebben met informatie en gesprekken over vruchtbaarheid. U kunt per uitspraak uit 6 opties kiezen. Geef aan hoezeer u het met deze uitspraken eens of oneens bent door het cijfer te omcirkelen bij het antwoord dat op u van toepassing is. Als u het zich niet kunt herinneren of als u geen van de antwoordopties geschikt vindt, omcirkel dan de uitspraak weet ik niet.

		Geheel mee oneens	Oneens	Niet mee oneens, niet mee eens	Eens	Geheel mee eens	Weet ik niet
1	Ik kreeg de mogelijkheid om vragen over vruchtbaarheid te stellen aan zorg-personeel (artsen, verpleegkundigen)	1	2	3	4	5	0
2	Ik heb zelf om informatie over vruchtbaarheid moeten vragen.	1	2	3	4	5	0
3	Ik heb informatie over vruchtbaarheid verkregen via andere bronnen, zoals internet, folders, andere ouders, familie, vrienden, kennissen	1	2	3	4	5	0
4	Ik vond het moment waarop gesproken werd over vruchtbaarheid een goed moment.	1	2	3	4	5	0
5	Ik vond het ondersteunende materiaal dat tijdens de uitleg gebruikt werd verduidelijkend.	1	2	3	4	5	0
6	Ik weet wat het risico voor mijn kind is op onvruchtbaarheid door de behandeling.	1	2	3	4	5	0
7	De uitleg over een mogelijk effect van de behandeling op de vruchtbaarheid was duidelijk	1	2	3	4	5	0
8	Ik heb zelf ook voldoende kunnen inbrengen tijde het gesprek/ de gesprekken over vruchtbaarheid	1	2	3	4	5	0
9	Ik heb belangrijke dingen gemist tijdens het gesprek/ de gesprekken	1	2	3	4	5	0
10	Ik had na afloop van het gesprek/de gesprekken nog steeds vragen over vruchtbaarheid.	1	2	3	4	5	0

		Geheel mee oneens	Oneens	Niet mee oneens, niet mee eens	Eens	Geheel mee eens	Weet ik niet
11	Ik heb uitleg gekregen over de mogelijkheden die er zijn om vruchtbaarheid te behouden	1	2	3	4	5	0
12	De uitleg over de behandelmogelijkheden was duidelijk	1	2	3	4	5	0
13	De voordelen van vruchtbaarheidsbehoudende behandelingen zijn besproken.	1	2	3	4	5	0
14	De nadelen van vruchtbaarheidsbehoudende behandelingen zijn besproken.	1	2	3	4	5	0
15	Er was ruimte om mee te beslissen over een eventuele behandeling voor vruchtbaarheidsbehoud.	1	2	3	4	5	0
16	Mijn behandelaars waren eerlijk en duidelijk over wat ik kon verwachten van een vruchtbaarheidsbehandeling.	1	2	3	4	5	0
17	Ik vond het belangrijk om informatie over vruchtbaarheid te krijgen	1	2	3	4	5	0
18	Toen ik de diagnose en behandeling hoorde, maakte ik mij zorgen om de vruchtbaarheid van mijn kind	1	2	3	4	5	0
19	Ik maak me op dit moment zorgen om de vruchtbaarheid van mijn kind	1	2	3	4	5	0
20	Als ik in de toekomst vragen heb over de vruchtbaarheid van mijn kind, weet ik hoe ik opnieuw een gesprek kan aanvragen.	1	2	3	4	5	0

De vragenlijst gaat door op de volgende bladzijde

Vruchtbaarheidsbehoud

5. Is er een mogelijkheid aangeboden voor een behandeling voor behoud van vruchtbaarheid?
   - ☐ Ja
   - ☐ Nee, einde van de vragenlijst

6. Welke vruchtbaarheidsbehoudende behandeling werd er aangeboden?
   - ☐ Zaad invriezen (semen cryopreservatie)
   - ☐ Zaadbal weefsel invriezen (testis biopsie)

7. Heeft u er uiteindelijk voor gekozen om deze vruchtbaarheidsbehoudende behandeling(en) te laten plaatsvinden?
   - ☐ Ja
   - ☐ Nee, wilt u aangeven waarom u ervoor koos om geen behandeling te laten plaatsvinden? (u kunt meerdere vakjes aankruisen)
     - ☐ Te ziek/belastend
     - ☐ Te jong
     - ☐ Dit voelde ongemakkelijk
     - ☐ Onzekerheden over het gebruik in de toekomst
     - ☐ Anders, namelijk……………………………………………………………………………
       ………………………………………………………………………………………………………
       ………………………………………………………………………………………………………
       ………………………………………………………………………………………………………

8. Heeft uw kind mee besloten of er een vruchtbaarheidsbehoudende behandeling zou plaatsvinden?
   - ☐ Ja
   - ☐ Nee

De vragenlijst gaat door op de volgende bladzijde

Kunt u aangeven in hoeverre u het eens bent met onderstaande stellingen? U kunt per uitspraak weer uit 6 opties kiezen. Geef aan hoezeer u het met deze uitspraken eens of oneens bent door het cijfer te omcirkelen bij het antwoord dat op u van toepassing is. Op deze manier geeft u aan wat u vindt van de beslissing die u genomen heeft. Als u het zich niet kunt herinneren of als u geen van de antwoordopties geschikt vindt, omcirkel dan de uitspraak weet ik niet.

		Geheel mee oneens	Oneens	Niet mee oneens, niet mee eens	Eens	Geheel mee eens	Weet ik niet
1	Ik vond het moeilijk om een keuze te moeten maken	1	2	3	4	5	0
2	Ik ervaarde stress voor het maken van een keuze	1	2	3	4	5	0
3	Ik heb kunnen kiezen zonder druk of beïnvloeding van anderen.	1	2	3	4	5	0
4	Ik heb de juiste beslissing gemaakt over vruchtbaarheidsbehoud.	1	2	3	4	5	0
5	Ik heb spijt van de beslissing die ik heb gemaakt	1	2	3	4	5	0
6	Ik zou nu een andere beslissing maken.	1	2	3	4	5	0

Dit is het einde van vragenlijst deel A. Deel B van de vragenlijst hoeft alleen ingevuld te worden als er geen gesprek over vruchtbaarheid is geweest

VRAGENLIJST DEEL B

Dit deel van de vragenlijst hoeft u alleen in te vullen als er geen gesprek over vruchtbaarheid is geweest. Als er wel een gesprek over vruchtbaarheid is geweest en u vragenlijst deel A heeft ingevuld, bent u klaar met deze vragenlijst.

Als er geen gesprek over vruchtbaarheid is geweest

Hieronder staan 5 uitspraken die te maken hebben met vruchtbaarheid. U kunt per uitspraak uit 6 opties kiezen. Geef aan hoezeer u het met deze uitspraken eens of oneens bent door het cijfer te omcirkelen bij het antwoord dat op u van toepassing is. Als u het zich niet kunt herinneren of als u geen van de antwoordopties geschikt vindt, omcirkel dan de uitspraak weet ik niet.

		Geheel mee oneens	Oneens	Niet mee oneens, niet mee eens	Eens	Geheel mee eens	Weet ik niet
1	Ik had graag informatie over vruchtbaarheid willen krijgen van betrokken zorg-personeel (artsen, verpleegkundigen)	1	2	3	4	5	0
2	Ik heb informatie over vruchtbaarheid verkregen via andere bronnen, zoals internet, andere ouders, familie, vrienden, kennissen	1	2	3	4	5	0
3	Toen ik de diagnose en behandeling hoorde, maakte ik mij zorgen om de vruchtbaarheid van mijn kind	1	2	3	4	5	0
4	Ik maak me op dit moment zorgen om de vruchtbaarheid van mijn kind	1	2	3	4	5	0
5	Ik weet hoe ik een gesprek kan aanvragen als ik (in de toekomst) vragen heb over de vruchtbaarheid van mijn kind	1	2	3	4	5	0

Dit is het einde van de vragenlijst. Hartelijk dank voor het invullen!

VRAGENLIJST 2:

Beste ……………………………….,

Je bent behandeld voor een Hodgkin Lymfoom en je doet mee aan een studie waarbij onderzocht wordt wat de gevolgen zijn van de behandeling van Hodgkin voor de vruchtbaarheid (het krijgen van kinderen).

Wij willen met deze vragenlijst onderzoeken hoe je de gesprekken die gingen over vruchtbaarheid vond. We hebben je ouder(s) ook vragen gesteld over deze gesprekken.

De vragenlijst mag samen met de vragenlijst die ingevuld is door je ouder(s) naar ons toe gestuurd worden via de post.

Wij willen je heel erg bedanken voor het invullen van deze vragenlijst!

Datum waarop deze vragenlijst is ingevuld: ……. - …... – 2023

VRAGENLIJST DEEL A

Gesprek(ken) over vruchtbaarheid

1. Is er voor zover je je kunt herinneren ooit met jou gesproken over vruchtbaarheid?
   - ☐ Ja
   - ☐ Nee, ga door naar deel B van deze vragenlijst

2. Wie heeft er met jou gesproken over vruchtbaarheid (je mag meerdere opties aanvinken)?
   - ☐ Ouders
   - ☐ Dokter of verpleegkundige (of een andere zorgverlener)

De vragenlijst gaat door op de volgende bladzijde

Hieronder staan 9 uitspraken die gaan over vruchtbaarheid. Je kan per uitspraak kiezen uit 6 antwoord-opties. Omcirkel het antwoord dat voor jou het beste bij de uitspraak past.

Als je je niet kunt herinneren hoe dit was of als je geen van de antwoordopties geschikt vindt, omcirkel dan de uitspraak weet ik niet

		Geheel mee oneens	Oneens	Niet mee oneens, niet mee eens	Eens	Geheel mee eens	Weet ik niet
1	Ik vond het belangrijk om informatie over vruchtbaarheid te krijgen	1	2	3	4	5	0
2	Ik vond het ongemakkelijk om te praten over mijn vruchtbaarheid	1	2	3	4	5	0
3	Ik kon vragen stellen aan artsen en/of verpleegkundigen	1	2	3	4	5	0
4	Ik vond het ondersteunende materiaal (zoals foto's of video's) dat tijdens de uitleg gebruikt werd verduidelijkend.	1	2	3	4	5	0
5	Ik heb duidelijke uitleg gekregen over mijn vruchtbaarheid	1	2	3	4	5	0
6	Ik heb informatie over vruchtbaarheid opgezocht op internet	1	2	3	4	5	0
7	Toen ik de diagnose en behandeling hoorde, maakte ik mij zorgen om mijn vruchtbaarheid	1	2	3	4	5	0
8	Ik maak me op dit moment zorgen om mijn vruchtbaarheid	1	2	3	4	5	0
9	Als ik in de toekomst vragen heb over mijn vruchtbaarheid, weet ik hoe ik een gesprek kan aanvragen.	1	2	3	4	5	0

Dit is het einde van vragenlijst deel A. Deel B van de vragenlijst hoeft alleen ingevuld te worden als er niet met jou gesproken is over vruchtbaarheid

VRAGENLIJST DEEL B

Dit deel van de vragenlijst hoeft je alleen in te vullen als er niet met jou gesproken is over vruchtbaarheid. Als er wel een gesprek over vruchtbaarheid is geweest, en je hebt vragenlijst deel A ingevuld, dan ben je klaar met deze vragenlijst.

Als er geen gesprek over vruchtbaarheid is geweest

Hieronder staan 5 uitspraken die te maken hebben met vruchtbaarheid. Je kan per uitspraak kiezen uit 6 antwoord-opties. Omcirkel het antwoord dat voor jou het beste bij de uitspraak past.

Als je je niet kunt herinneren hoe dit was of als je geen van de antwoordopties geschikt vindt, omcirkel dan de uitspraak weet ik niet

		Geheel mee oneens	Oneens	Niet mee oneens, niet mee eens	Eens	Geheel mee eens	Weet ik niet
1	Ik had het graag informatie willen krijgen over vruchtbaarheid	1	2	3	4	5	0
2	Ik heb informatie over vruchtbaarheid opgezocht op internet	1	2	3	4	5	0
3	Toen ik de diagnose en behandeling hoorde, maakte ik mij zorgen om mijn vruchtbaarheid	1	2	3	4	5	0
4	Ik maak me op dit moment zorgen om mijn vruchtbaarheid	1	2	3	4	5	0
5	Als ik in de toekomst vragen heb over mijn vruchtbaarheid, weet ik hoe ik een gesprek kan aanvragen.	1	2	3	4	5	0

Dit is het einde van de vragenlijst. Bedankt voor het invullen!

EENMALIG VRAGENLIJST ONDERZOEK

Geachte ouder(s),

Uw kind kreeg de diagnose Hodgkin Lymfoom. Uw kind doet mee aan een vruchtbaarheidsstudie, de ‘fertility add-on’ studie die onderdeel is van de EuroNET-PHL-C2 studie. U heeft de informatie brief gelezen en aangegeven dat u mee wilt doen met een eenmalig vragenlijst onderzoek over vruchtbaarheid en de behandeling van Hodgkin.

De vragenlijst is bijgevoegd bij deze brief. U kunt deze ingevuld retour sturen aan de onderzoekers in het Prinses Maxima Centrum via de meegestuurde antwoordenvelop.

U kunt bij vragen of opmerkingen contact opnemen met de onderzoekers via:
k.c.e.drechsel-3@prinsesmaximacentrum.nl (arts-onderzoeker Katja Drechsel)

Wij willen u hartelijk danken voor uw deelname!

Dr. Margreet Veening, kinderarts-oncoloog
Drs. Katja Drechsel, arts-onderzoeker

VRAGENLIJST: OUDERS/VERZORGERS

Datum waarop deze vragenlijst is ingevuld: ……. - …… – 2023

VRAGENLIJST DEEL A

Gesprek(ken) over vruchtbaarheid

1. Is er ooit met u gesproken over de (toekomstige) vruchtbaarheid van uw kind?
    - ☐ Ja
    - ☐ Nee, ga door naar deel B van deze vragenlijst

2. In welke fases tijdens de behandeling is er gesproken over (toekomstige) vruchtbaarheid? (u kunt meerdere vakjes aankruisen)
    - ☐ Bij het diagnose-gesprek
    - ☐ Na het diagnose gesprek, maar voor start van de behandeling
    - ☐ Tijdens de behandeling
    - ☐ Na afronding van de behandeling

3. Met wie heeft u binnen het ziekenhuis over vruchtbaarheid gesproken? (u kunt meerdere vakjes aankruisen)
    - ☐ Behandelend arts
    - ☐ Verpleegkundig specialist
    - ☐ Uroloog
    - ☐ Anders, namelijk:
      ………………………………………………………………………………………………………
      ……………………………………………………………………………………………………..

4. Is uw zoon bij de counselingsgesprekken (die gingen over vruchtbaarheid) aanwezig geweest ?
    - ☐ Ja
    - ☐ Nee, kunt u aangeven waarom uw zoon niet betrokken is geweest bij de gesprekken die gingen over vruchtbaarheid? (u kunt meerdere vakjes aankruisen)
        - ☐ Te jong
        - ☐ Te ziek/belastend
        - ☐ Geen interesse/behoefte
        - ☐ Dit voelde ongemakkelijk
        - ☐ Anders, namelijk……………………………………………………………………………
          ……………………………………………………………………………………………….
          …………………………………………………………………………………………………
          …………………………………………………………………………………………………

Hieronder staan 20 uitspraken die te maken hebben met informatie en gesprekken over vruchtbaarheid. U kunt per uitspraak uit 6 opties kiezen. Geef aan hoezeer u het met deze uitspraken eens of oneens bent door het cijfer te omcirkelen bij het antwoord dat op u van toepassing is. Als u het zich niet kunt herinneren of als u geen van de antwoordopties geschikt vindt, omcirkel dan de uitspraak weet ik niet.

		Geheel mee oneens	Oneens	Niet mee oneens, niet mee eens	Eens	Geheel mee eens	Weet ik niet
1	Ik kreeg de mogelijkheid om vragen over vruchtbaarheid te stellen aan zorg-personeel (artsen, verpleegkundigen)	1	2	3	4	5	0
2	Ik heb zelf om informatie over vruchtbaarheid moeten vragen.	1	2	3	4	5	0
3	Ik heb informatie over vruchtbaarheid verkregen via andere bronnen, zoals internet, folders, andere ouders, familie, vrienden, kennissen	1	2	3	4	5	0
4	Ik vond het moment waarop gesproken werd over vruchtbaarheid een goed moment.	1	2	3	4	5	0
5	Ik vond het ondersteunende materiaal dat tijdens de uitleg gebruikt werd verduidelijkend.	1	2	3	4	5	0
6	Ik weet wat het risico voor mijn kind is op onvruchtbaarheid door de behandeling.	1	2	3	4	5	0
7	De uitleg over een mogelijk effect van de behandeling op de vruchtbaarheid was duidelijk	1	2	3	4	5	0
8	Ik heb zelf ook voldoende kunnen inbrengen tijdens het gesprek/ de gesprekken over vruchtbaarheid	1	2	3	4	5	0
9	Ik heb belangrijke dingen gemist tijdens het gesprek/ de gesprekken	1	2	3	4	5	0
10	Ik had na afloop van het gesprek/de gesprekken nog steeds vragen over vruchtbaarheid.	1	2	3	4	5	0

		Geheel mee oneens	Oneens	Niet mee oneens, niet mee eens	Eens	Geheel mee eens	Weet ik niet
11	Ik heb uitleg gekregen over de mogelijkheden die er zijn om vruchtbaarheid te behouden	1	2	3	4	5	0
12	De uitleg over de behandelmogelijkheden was duidelijk	1	2	3	4	5	0
13	De voordelen van vruchtbaarheidsbehoudende behandelingen zijn besproken.	1	2	3	4	5	0
14	De nadelen van vruchtbaarheidsbehoudende behandelingen zijn besproken.	1	2	3	4	5	0
15	Er was ruimte om mee te beslissen over een eventuele behandeling voor vruchtbaarheidsbehoud.	1	2	3	4	5	0
16	Mijn behandelaars waren eerlijk en duidelijk over wat ik kon verwachten van een vruchtbaarheidsbehandeling.	1	2	3	4	5	0
17	Ik vond het belangrijk om informatie over vruchtbaarheid te krijgen	1	2	3	4	5	0
18	Toen ik de diagnose en behandeling hoorde, maakte ik mij zorgen om de vruchtbaarheid van mijn kind	1	2	3	4	5	0
19	Ik maak me op dit moment zorgen om de vruchtbaarheid van mijn kind	1	2	3	4	5	0
20	Als ik in de toekomst vragen heb over de vruchtbaarheid van mijn kind, weet ik hoe ik opnieuw een gesprek kan aanvragen.	1	2	3	4	5	0

De vragenlijst gaat door op de volgende bladzijde

Vruchtbaarheidsbehoud

5. Is er een mogelijkheid aangeboden voor een behandeling voor behoud van vruchtbaarheid?
   - ☐ Ja
   - ☐ Nee, einde van de vragenlijst

6. Welke vruchtbaarheidsbehoudende behandeling werd er aangeboden?
   - ☐ Zaad invriezen (semen cryopreservatie)
   - ☐ Zaadbal weefsel invriezen (testis biopsie)

7. Heeft u er uiteindelijk voor gekozen om deze vruchtbaarheidsbehoudende behandeling(en) te laten plaatsvinden?
   - ☐ Ja
   - ☐ Nee, wilt u aangeven waarom u ervoor koos om geen behandeling te laten plaatsvinden? (u kunt meerdere vakjes aankruisen)
     - ☐ Te ziek/belastend
     - ☐ Te jong
     - ☐ Dit voelde ongemakkelijk
     - ☐ Onzekerheden over het gebruik in de toekomst
     - ☐ Anders, namelijk…………………………………………………………………………
       ……………………………………………………………………………………………………
       ……………………………………………………………………………………………………
       ……………………………………………………………………………………………………

8. Heeft uw kind mee besloten of er een vruchtbaarheidsbehoudende behandeling zou plaatsvinden?
   - ☐ Ja
   - ☐ Nee

De vragenlijst gaat door op de volgende bladzijde

Kunt u aangeven in hoeverre u het eens bent met onderstaande stellingen? U kunt per uitspraak weer uit 6 opties kiezen. Geef aan hoezeer u het met deze uitspraken eens of oneens bent door het cijfer te omcirkelen bij het antwoord dat op u van toepassing is. Op deze manier geeft u aan wat u vindt van de beslissing die u genomen heeft. Als u het zich niet kunt herinneren of als u geen van de antwoordopties geschikt vindt, omcirkel dan de uitspraak weet ik niet.

		Geheel mee oneens	Oneens	Niet mee oneens, niet mee eens	Eens	Geheel mee eens	Weet ik niet
1	Ik vond het moeilijk om een keuze te moeten maken	1	2	3	4	5	0
2	Ik ervaarde stress voor het maken van een keuze	1	2	3	4	5	0
3	Ik heb kunnen kiezen zonder druk of beïnvloeding van anderen.	1	2	3	4	5	0
4	Ik heb de juiste beslissing gemaakt over vruchtbaarheidsbehoud.	1	2	3	4	5	0
5	Ik heb spijt van de beslissing die ik heb gemaakt	1	2	3	4	5	0
6	Ik zou nu een andere beslissing maken.	1	2	3	4	5	0

Dit is het einde van vragenlijst deel A. Deel B van de vragenlijst hoeft alleen ingevuld te worden als er geen gesprek over vruchtbaarheid is geweest

VRAGENLIJST DEEL B

Dit deel van de vragenlijst hoeft u alleen in te vullen als er geen gesprek over vruchtbaarheid is geweest. Als er wel een gesprek over vruchtbaarheid is geweest en u vragenlijst deel A heeft ingevuld, bent u klaar met deze vragenlijst.

Als er geen gesprek over vruchtbaarheid is geweest

Hieronder staan 5 uitspraken die te maken hebben met vruchtbaarheid. U kunt per uitspraak uit 6 opties kiezen. Geef aan hoezeer u het met deze uitspraken eens of oneens bent door het cijfer te omcirkelen bij het antwoord dat op u van toepassing is. Als u het zich niet kunt herinneren of als u geen van de antwoordopties geschikt vindt, omcirkel dan de uitspraak weet ik niet.

		Geheel mee oneens	Oneens	Niet mee oneens, niet mee eens	Eens	Geheel mee eens	Weet ik niet
1	Ik had graag informatie over vruchtbaarheid willen krijgen van betrokken zorg-personeel (artsen, verpleegkundigen)	1	2	3	4	5	0
2	Ik heb informatie over vruchtbaarheid verkregen via andere bronnen, zoals internet, andere ouders, familie, vrienden, kennissen	1	2	3	4	5	0
3	Toen ik de diagnose en behandeling hoorde, maakte ik mij zorgen om de vruchtbaarheid van mijn kind	1	2	3	4	5	0
4	Ik maak me op dit moment zorgen om de vruchtbaarheid van mijn kind	1	2	3	4	5	0
5	Ik weet hoe ik een gesprek kan aanvragen als ik (in de toekomst) vragen heb over de vruchtbaarheid van mijn kind	1	2	3	4	5	0

Dit is het einde van de vragenlijst. Hartelijk dank voor het invullen!
