# Supplementary material for: Fertility-Preserving Treatments and Patient- and Parental Satisfaction on Fertility Counseling in a Cohort of Newly Diagnosed Boys and Girls with Childhood Hodgkin Lymphoma
Source: Cancers (Basel). 2024 May 31;16(11):2109. doi: 10.3390/cancers16112109 (PMC11171249; doi:10.3390/cancers16112109)
Supplement: Supplementary file 1 [file cancers-16-02109-s001.zip › Supplementary Table S1.pdf]

Supplementary Table S1: Reasons for not choosing a fertility preserving treatment as reported by research nurse on the case-report form

	MALES		FEMALES		
	Recorded reasons for not performing		Recorded reasons for not performing		
	Semen cryopreservation		Ovariopexy	OTC	Oocyte cryopreservation
	53 patients*		102 patients	93 patients	100 patients
Tanner stage <4 and/or testicular volume <15 ml as measured by Prader orchidometer)	35 (66.0%)	Not suggested by physician	29 (28.4%)	37 (39.8%)	39 (39.0%)
Physically not able	11 (20.7%)	Patient/parental refusion	11 (10.8%)	13 (14.0%)	13 (13.0%)
Emotionally not able	8 (15.1%)	Not a standard procedure at the institution		8 (8.6%)	10 (10.0%)
Patient/parental refusion	5 (9.4%)	Considered too young		3 (3.2%)	4 (4.0%)
Not suggested by physician	2 (3.8%)	Low risk	18 (17.6%)	18 (19.4%)	17 (17.0%)
Unknown	2 (3.8%)	Unknown	13 (12.7%)	14 (15.1%)	17 (17.0%)
		Not applicable	37 (36.3%)		

OTC; ovarian tissue cryopreservation.
Number do not add up as it was possible to select multiple answer options.
*semen collection was expected based on tanner stage and/or testicular volume in 9 boys who did not cryopreserve semen. Among these boys, recorded reasons for not performing semen cryopreservation included physically not able (n=1), emotionally not able (n=1), physically and emotionally noy able (n=1) patient/parental refusal (n=4) not suggested by physician (n=1) and unknown (n=1).
