# Supplementary material for: Fertility-Preserving Treatments and Patient- and Parental Satisfaction on Fertility Counseling in a Cohort of Newly Diagnosed Boys and Girls with Childhood Hodgkin Lymphoma
Source: Cancers (Basel). 2024 May 31;16(11):2109. doi: 10.3390/cancers16112109 (PMC11171249; doi:10.3390/cancers16112109)
Supplement: Supplementary file 1 [file cancers-16-02109-s001.zip › Supplementary Table S2.pdf]

Supplementary Table S2: Statements on fertility counseling answered by those who reported to have had no conversation about fertility

	Parents (n=1)	Patients (n=3)
Had wished to receive information	Agree	33% Disagree<br>33% Neutral<br>33% Agree
Obtained information from other sources	I don't know	50% Disagree<br>50% Neutral<br>*1 not answered*
Felt concerned about my (child's) fertility at time of diagnosis	Strongly agree	50% Disagree<br>50% Neutral<br>*1 not answered*
I am currently concerned about my (child's) fertility	Strongly agree	50% Disagree<br>50% Neutral<br>*1 not answered*
I know how to request (future) consultation on fertility	I don't know	50% Agree<br>50% Strongly agree<br>*1 not answered*
